# Beyond Reinforcement: Collagen–Inorganic Composites as a Roadmap for Next-Generation Biomaterials

**DOI:** 10.1021/acsmaterialsau.5c00192

**Published:** 2026-01-02

**Authors:** Marcelo Assis, Giovanna A. Grasser, Mirian Bonifacio, Karolyne S. J. Sousa, Amanda de Souza, Anna Rafaela Cavalcante Braga, Ana Claudia Muniz Renno

**Affiliations:** † Department of Biosciences, 28105 Universidade Federal de São Paulo (UNIFESP), 11015-020 Santos, Brazil; ‡ CDMF, 67828 Universidade Federal de São Carlos (UFSCar), 13565-905 São Carlos, Brazil; § Nutrition and Food Service Research Center, Universidade Federal de São Paulo (UNIFESP), 11015-020 Santos, Brazil; ∥ Department of Chemical Engineering, Universidade Federal de São Paulo (UNIFESP), 09972-270 Diadema, Brazil

**Keywords:** collagen-based biomaterials, composites, inorganic materials, tissue engineering, regenerative medicine, interface engineering, bioactivity modulation, scaffold architecture

## Abstract

The convergence of materials science and biology has reshaped the design of biomaterials, exposing both new opportunities and unresolved challenges. Among natural polymers, collagen remains a cornerstone due to its biocompatibility and structural affinity with the extracellular matrix. However, its intrinsic mechanical weakness, rapid degradation, and limited bioactivity restrict its clinical potential. The incorporation of inorganic phases—carbon nanostructures, metallic nanoparticles, or functional oxides—has emerged as a route to overcome these limitations and introduce new functionalities such as antimicrobial protection, osteoconductivity, electrical responsiveness, and stimuli sensitivity. Yet, this hybridization introduces complex interfacial phenomena that demand careful architectural and chemical control. The spatial organization of pores, fibers, and surface topographies governs nutrient diffusion and cell alignment, while interface chemistry dictates stability, degradation, and biological signaling. Despite significant progress, reproducibility and long-term safety remain inconsistent across studies, hindered by variations in collagen source, particle distribution, and cross-linking strategies. Beyond empirical formulation, future progress requires mechanism-guided design frameworks that link composition, structure, and function to predictable biological outcomes. This review critically examines advances in collagen–inorganic composites, highlighting key structure–property–function relationships, manufacturing strategies, and translational barriers. By mapping trends through bibliometric analysis and synthesizing evidence from recent studies, it outlines a roadmap toward reproducible, multifunctional, and clinically relevant collagen-based biomaterials.

## Graphical abstract

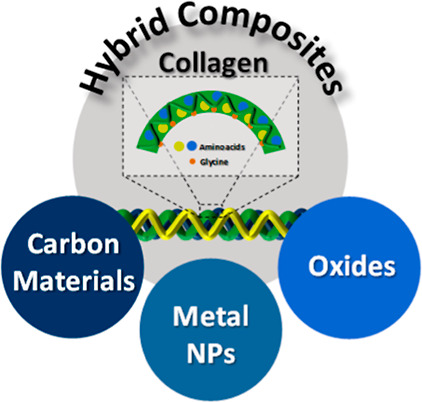

## 1 Introduction

The convergence of materials and life sciences, coupled with allied technologies, has significantly advanced our understanding at the atomic and molecular levels, unraveling the intricate connections among chemical compositions, structures, and properties of materials. Yet, as this molecular-level understanding extends into living systems, translating these insights into predictable biological outcomes remains one of the most persistent challenges in modern materials science. The complexity of biological environments—dynamic, heterogeneous, and often patient-specific—frequently undermines the linear assumptions derived from conventional materials engineering. As this knowledge extends into living systems, navigating the complexities of interactions between materials and their intended targets poses a formidable challenge in the realm of new technological developments. While synthetic chemistry and materials engineering have propelled fundamental synthetic technologies for constructing novel materials, the burgeoning field of biology is charting novel paths to explore chemical space, uncovering unique chemical interactions not easily accessible to synthetic chemists and rapidly investigating a diverse array of molecular structures. This convergence forces materials scientists to integrate biological feedback into design strategies, promoting a more adaptive and mechanism-oriented understanding of material–cell interactions.

This contemporary landscape has ushered in a new era of synthetic biological materials called biomaterials. Traditionally associated with healthcare applications, disease treatment, injury management, diagnostics, and more recently, biomaterials have become pivotal in addressing various health-related challenges. However, the rapid expansion of this field also exposes a central paradox: innovation is advancing faster than understanding. The potential applications for biomaterials are diverse, with a noticeable surge in investments and research endeavors, fueled by advancements in nanotechnology and a globally aging population.^1,2^ This surge reflects an unconscious drive toward discovering innovative solutions to human health challenges. At the heart of any approach to acquiring new biomaterials lies predictive design and rapid evaluation, aligning closely with the fundamental principle of addressing specific needs within the biomaterial landscape. Yet, predictive tools and accelerated screening must be balanced with realistic biological testing to avoid premature assumptions about biocompatibility or stability.

Various matrices show promise for their design and development of biomaterials, varying in type according to their function and property. Within the classification of materials, metallic biomaterials are most explored for the development of prosthetics, screws, and pins, commonly used in orthopedics.^3–5^ Ceramic biomaterials can be found, especially in the dental field, to mimic dental structures and regenerate bones.^6–8^ Polymeric biomaterials present themselves as the most versatile material for their application (Figure 1).^9^ They can be used for tissue engineering, drug delivery systems, and developing reinforcing structures such as stents and others.^10–12^ Among the classes of polymeric biomaterials, natural biopolymers stand out due to their high biocompatibility, bioabsorption and manufacturing versatility.^13,14^ Notable among them are chitosan, alginate, collagen, cellulose, etc.^15–18^ Among these biopolymers, collagen stands out for its use in tissue engineering due to its ability to mimic the extracellular matrix (ECM), providing a foundation for cell proliferation and angiogenesis.^15,19,20^ Beyond structural mimicry, collagen actively mediates biochemical communication with cells, influencing adhesion, migration, and differentiation -features rarely replicated by synthetic polymers.

**Figure 1:**
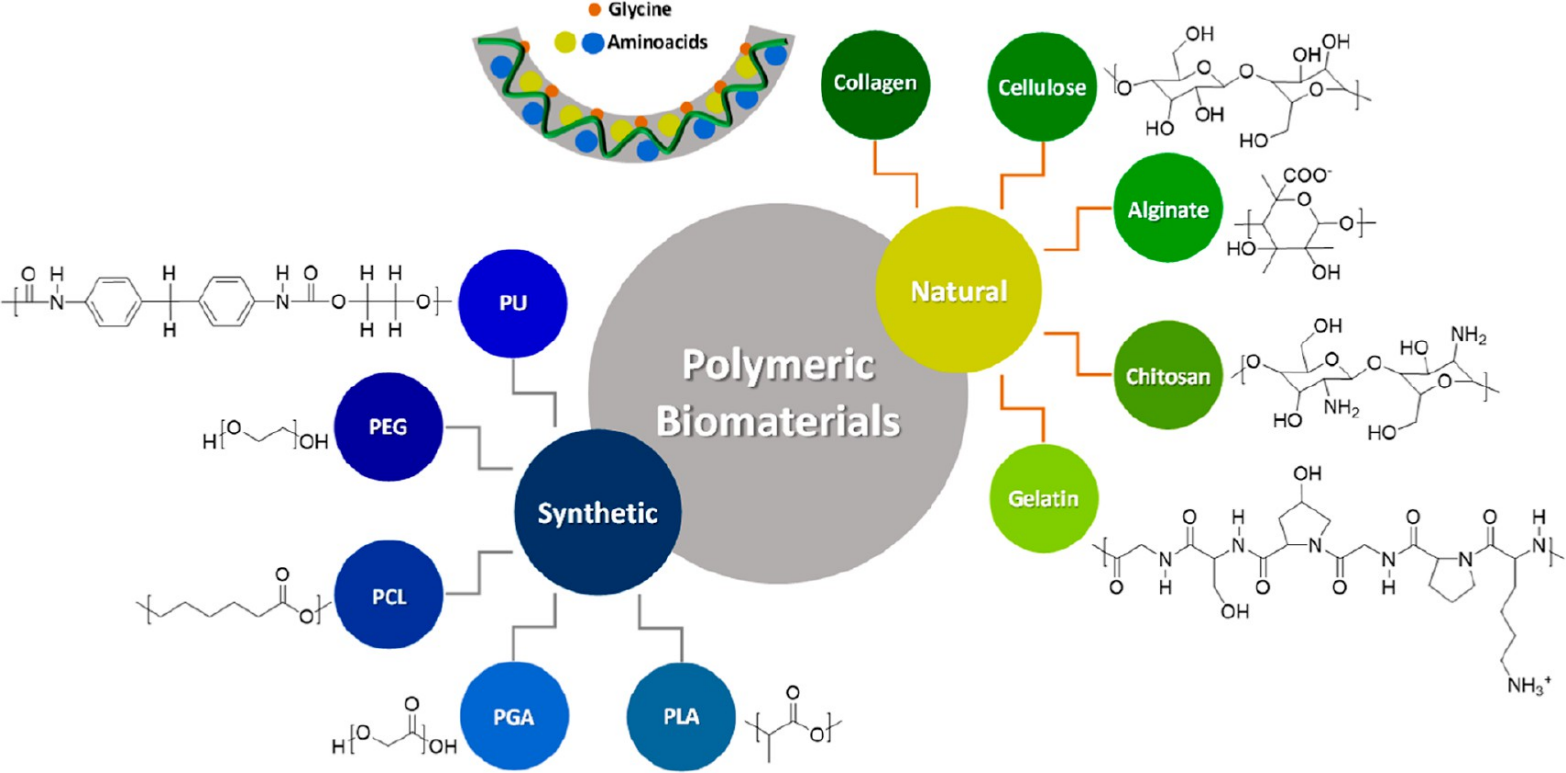
Most common polymers for biomaterials manufacturing.

The last class of materials is the composites, where two or more different materials (of the same type or different types) are combined. Composites biomaterial engineering is primarily used to achieve synergy between both structures, harnessing the best properties of these materials.^21^ Despite being promising, composite biomaterials engineering is a complex science due to the interaction between two or more materials that compose it. Thus, factors such as dispersion, degradation, electrostatic interactions, and affinity between materials must be considered to obtain a composite biomaterial with amplified properties.^22,23^ Small variations in particle size, distribution, or surface energy can drastically alter the mechanical behavior and biological response of composites, emphasizing how critical interface control has become.

Collagen occupies a central position in the development of composite biomaterials because it provides a natural and biologically familiar framework that closely resembles the extracellular matrix. Its intrinsic ability to support cell adhesion, proliferation, and communication makes it an ideal base for constructing scaffolds intended for tissue repair. However, in its pure form, collagen presents critical limitations. The incorporation of inorganic particles into collagen matrices has emerged as a strategy to overcome these restrictions and, at the same time, introduce new functionalities. Depending on their nature, these inorganic components can strengthen the matrix, regulate its degradation profile, and contribute properties such as antimicrobial protection, osteoconductivity, angiogenic stimulation, electrical conductivity, or responsiveness to external stimuli. Nonetheless, this integration demands a balance between reinforcement and biological compatibility, as poorly engineered interfaces can generate local stress points, irregular degradation, or inflammatory responses. The resulting synergy transforms collagen from a passive structural substrate into a dynamic and multifunctional platform, capable of guiding complex biological processes and meeting the demands of advanced tissue engineering.

Building on this rationale, it becomes clear that the development of collagen-based composites cannot rely on a purely additive perspective but requires a deliberate and integrated design strategy. The engineering of biomaterials must consider not only the immediate reinforcement or functionality imparted by the inorganic phase, but also the broader implications for cellular regeneration, tissue integration, and long-term performance. Too often, studies highlight isolated benefits while overlooking aspects such as degradation pathways, immune interactions, or manufacturability. Therefore, predictive modeling and standardized evaluation protocols are essential to ensure reproducibility and reliability across laboratories. A more holistic framework is therefore needed—one that spans the entire lifecycle of the material, from conceptual design and fabrication to biological interaction and clinical translation—ensuring that collagen composites evolve from promising prototypes into reliable technologies for regenerative medicine.

The current review aims to present results found in the literature about the production of composite materials obtained in a collagen matrix with inorganic materials, demonstrating their advantages and disadvantages through generating and investigating a bibliometric map of articles using VosViewer software (Figure 2). Manufacturing methods and their applications will be discussed to provide an overview of the use of these composites in tissue engineering.

**Figure 2:**
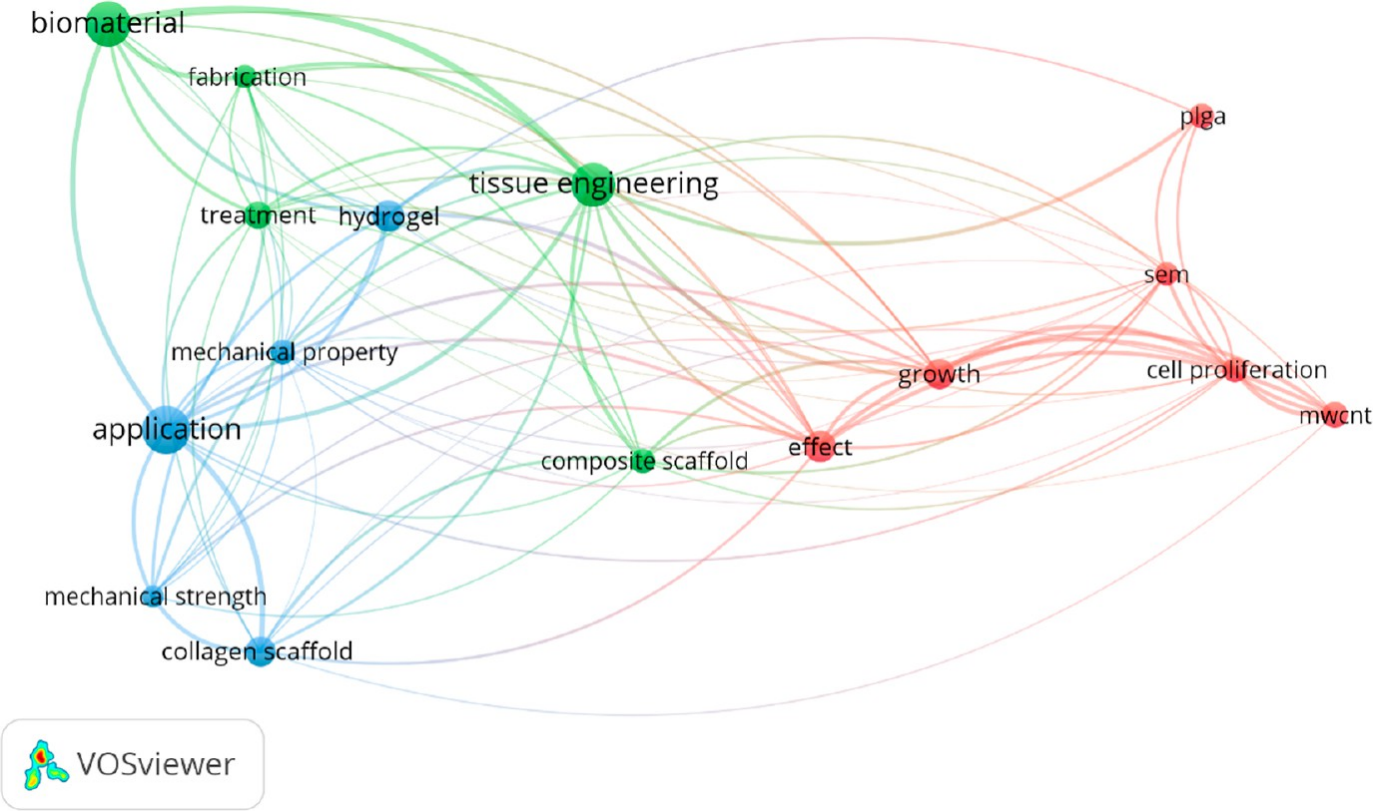
Density visualization map results from the keywords applied in the search conducted in the present work using VosViewer software.

## 2 Tissue Engineering

Every year, millions of patients experience total or partial damage to their organs and tissues, making them potential candidates for studies in the field of regenerative medicine. This gave rise to the field of tissue engineering, aiming to develop functional tissues capable of regenerating and/or improving damaged tissues, requiring contributions from various fields. Its origin dates back to the 1980s, making it a young and promising science in line with current contexts.^24^ This research area is evolving exponentially, focusing mainly on developing decellularized matrices, artificial tissues, and scaffolds. Its primary focus includes creating substitutes for organs, tissues, and cellular structures for clinical applications.^25^

In the context of tissue engineering, the development of biomaterials plays a crucial role. Biomaterials are substances that interact in a controlled manner with biological systems and can be used to create three-dimensional scaffolds, implantable devices, and vehicles for delivering of cells, drugs, and growth factors.^11,26^ The advantage of biomaterials lies in their ability to provide temporary structural support, promote cell adhesion, and, in some cases, be entirely absorbed by the body.^27,28^ Scaffolds are one of the most studied types of biomaterials, consisting of a three-dimensional platform that can mimic the ECM and provide mechanical, spatial, and biological signals to regulate and guide cellular responses.^29–31^

To give an idea of the economic and social context of skin tissue engineering, for example, it is estimated that around 2% of the global population experiences chronic and challenging-to-heal wounds during their lifetime,^32^ with approximately 7 million patients suffering from acute and chronic wounds in the United States alone, leading to annual expenditures of about $25 billion on treatment.^33^ In Europe, the cost of acute and chronic skin wounds exceeds €8 billion annually for approximately 2 million people.^34^ Consequently, the global wound care market continues to expand, projected to exceed $18.5 billion in the coming years. These costs encompass supplies and dressings (15–20%), nursing time (30–35%), and hospitalization (over 50%).^35^ To address this economic burden, innovative biomaterials for skin regeneration offer a promising avenue to reduce healthcare expenses related to wound care, hospitalization, procurement, and complications. Additionally, developing on-site production capabilities in hospital settings can yield cost savings by eliminating the need for complex centralized manufacturing processes (Figure 3).

**Figure 3:**
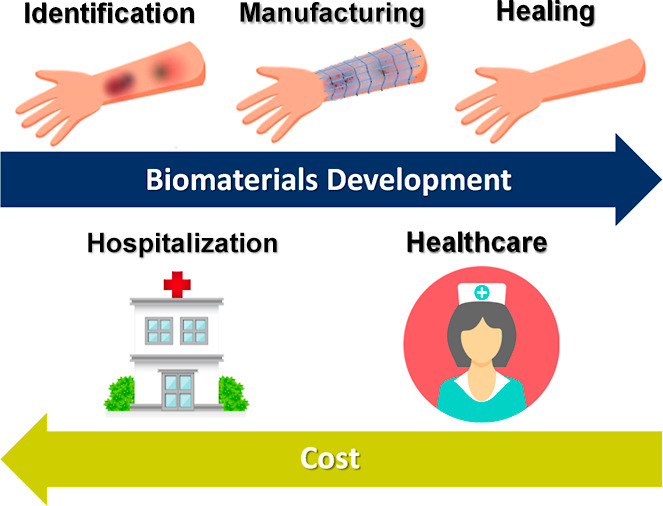
Impact of advanced biomaterials on healthcare costs: Reduction in treatment time, lower incidence of complications, and decrease in hospitalization and reintervention rates.

However, using biomaterials in tissue engineering also faces significant challenges, such as high costs associated with research and development, ethical issues related to the source and manipulation of biological materials, and scientific challenges to ensure the long-term functionality of generated tissues. According to Williams, tissue engineering has not yet fulfilled its initial promises due to the high required investments, regulation, ethics, bioprocessing, and infrastructure.^36^ The pursuit of advancements in this field requires a delicate balance between scientific innovation, ethical considerations, and economic viability, highlighting the complexity and intrinsic promise of tissue engineering.

## 3 Collagen

Collagen is a natural protein found in the connective tissues of the human body, such as skin, bones, tendons, and cartilage, representing approximately 25% of the total dry weight of mammals.^37^ Various collagen types have been characterized, all displaying a typical triple-helix structure, with most of its production in connective tissues being carried out by fibroblasts (Figure 4). The various types of collagen, like I, II, III, V, and XI, are recognized for their ability to create collagen fibers.^20^ Each collagen molecule comprises three α chains, and their molecular structure governs their assembly.^19^ Collagen’s inherent biological compatibility, stemming from its natural occurrence within the body, enhances its compatibility and reduces the likelihood of eliciting negative immune responses. Consequently, collagen stands out as a highly promising natural biopolymer for constructing of scaffolds in tissue engineering.

**Figure 4:**
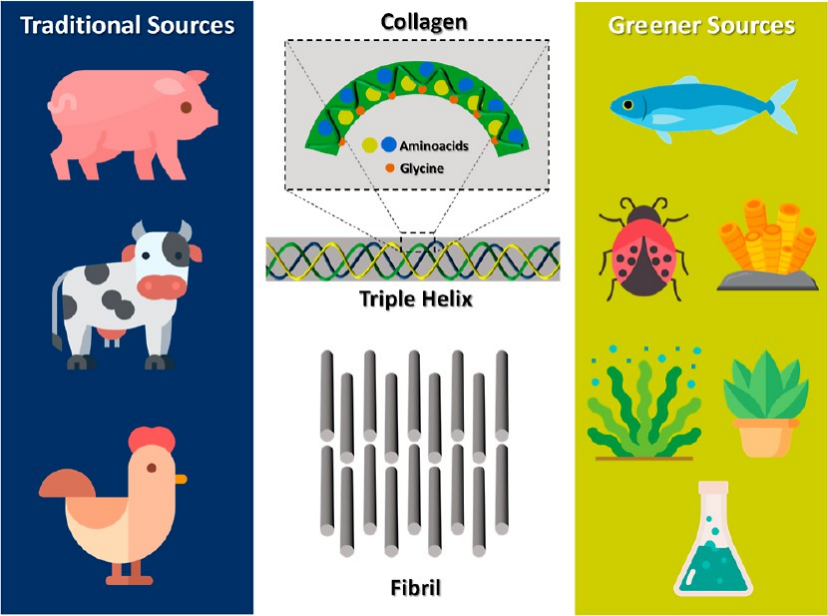
Structure of collagen and its sources.

Its molecular structure provides physical and mechanical support to tissues, a crucial factor in tissue engineering, aiding in forming and maintaining the necessary three-dimensional structure for cell growth and tissue function.^38^ Collagen possesses specific binding sites that facilitate cell adhesion and migration, essential for cellular growth and proliferation in tissue engineering processes.^39^ Additionally, collagen is biodegradable, meaning it can be gradually decomposed by the body as new tissues form, ensuring efficient replacement of the implanted material by the body’s tissues.^40^

Collagen can be obtained from various sources, commonly of animal origin, such as cattle, pigs, and poultry. However, alternatives that do not involve animal farming are being explored for more sustainable, environmentally friendly options. This includes fish collagen from the scales and skin and, studies on extracting collagen from insects.^41,42^ Furthermore, initial research investigates the feasibility of obtaining collagen from algae and marine sponges,^43^ while genetic engineering and other advanced techniques aim to create synthetic plant-based collagen.^44^ Regarding sustainability and environmental impact, collagen from plant or synthetic sources is generally considered greener, avoiding concerns associated with intensive animal farming. In addition, plant-derived recombinant human collagen has shown reduced immunogenicity and inflammatory response compared to bovine collagen, while synthetic collagen-mimetic systems offer high reproducibility and precise molecular control with minimal immune activation.^45–48^ However, it is crucial to note that plant-based or synthetic alternatives are still in the early stages of development compared to traditional animal-derived collagen sources.

The collagen source significantly influences its physical, chemical, and biological properties due to amino acid composition and molecular structure variations.^49^ Collagen from different animal sources, such as cattle, pigs, poultry, or fish, exhibits variations in molecular size, fibril organization, and molecular cross-linking patterns.^50–52^ These structural differences can impact collagen’s mechanical strength, flexibility, and thermal stability. Additionally, the specific amino acid composition, such as the higher presence of hydroxyproline in marine collagen, can influence biological properties, including the ability to promote cell regeneration and interactions with cells.^53,54^ Collagen derived from plant sources or produced synthetically may present distinct characteristics compared to animal-derived counterparts.^55^ Genetic engineering can selectively modulate amino acid composition to meet specific requirements, while plant sources offer potentially more sustainable alternatives, with implications for biocompatibility and clinical applications.

### 3.1 Manufacture of Collagen Scaffolds

Collagen is versatile in its application in tissue engineering, being processed into various forms such as gels, films, membranes, and fibers to meet specific needs across different applications. Each of these forms has distinct characteristics that are applied in unique ways. Collagen-based gels are often employed to create three-dimensional structures that support cellular growth in in vitro cultures.^56^ Conversely, collagen films are used as coatings; they can provide mechanical support and specific interactions with cells.^57,58^ Collagen membranes find application in diverse contexts, including tissue repair, organ regeneration, and wound closure.^59^ Collagen fibers, on the other hand, are utilized in tissues requiring greater mechanical strength, such as tendons and ligaments.^60^

The production of different forms of collagen for tissue engineering applications involves distinct processes, each adapted to meet specific properties (Figure 5). The process of obtaining collagen gels, begins with the extraction, purification, and treatment with acids to enhance collagen solubility. After that, collagen can undergo the gelation process by adjusting variables like pH and temperature to create consistent gels.^61^ These gels can also undergo physical processes such as freeze-drying, resulting in solvent-free 3D aerogels. From an architectural standpoint, the control of gel porosity, alignment, and density is crucial, as it defines nutrient diffusion and guides cell organization—reflecting the first design principle discussed earlier. The combination of different polymers, such as alginate and collagen, is explored too, to obtain hydrogels.^62^ Such hybrid formulations require special attention to the interface between phases: mismatched cross-linking densities or incompatible swelling behaviors can weaken the overall structure or alter cell–matrix communication. Careful interface engineering therefore becomes essential to maintain the integrity and functional balance of the composite.

**Figure 5:**
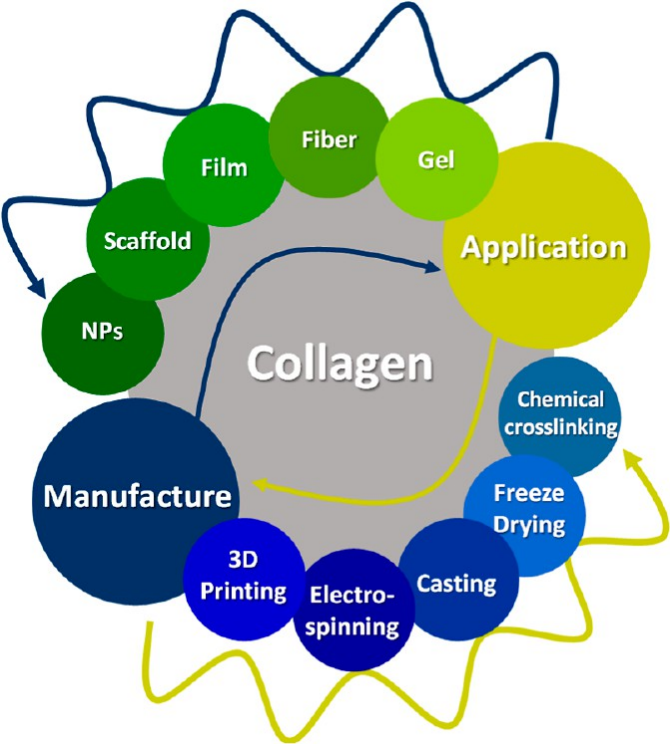
Manufacture and applications form of collagen-based biomaterials.

Collagen films, on the other hand, require the preparation of a collagenous solution, often supplemented with cross-linking agents to impart specific properties.^63,64^ This solution is then shaped into the desired form and subjected to drying or coagulation processes to form the collagen film. Film uniformity and interfacial cohesion are critical parameters; irregular solvent evaporation or uncontrolled cross-linking can generate heterogeneous surfaces that compromise both mechanical stability and biological performance. Consequently, optimizing the interface between collagen and any incorporated bioactive phase is as decisive as adjusting its chemical formulation.

In the realm of collagen membranes, the electrospinning technique is commonly employed.^65^ This technique involves forming ultrafine fibers by applying an electric field. Alternatively, membranes can be obtained by precipitating collagen from solutions, resulting in membranes with different characteristics. The micro- and nanoscale architecture generated through electrospinning offers direct control over pore interconnectivity and fiber alignment, parameters that modulate cell migration and tissue integration. However, poor control of fiber junctions or solvent residues can compromise interfacial adhesion between layers, affecting long-term stability. Collagen fibers, in turn, can be produced through wet or dry spinning processes, depending on processing conditions.^66^ The pulling through a matrix technique is often used to guide the fibers and obtain specific characteristics. In both spinning and electrospinning, tuning the molecular alignment and surface roughness of fibers governs not only tensile strength but also the way cells anchor and communicate—again emphasizing the interplay between architecture and interface.

Collagen 3D printing represents an innovative approach to tissue engineering.^67^ This technique is frequently used to manufacture three-dimensional scaffolds that mimic tissue architecture. Preparing a collagen-based ink, usually through a collagenous hydrogel combined with cells and other biomaterials, enables the controlled deposition of successive layers to build the final structure.^68^ Here, the printing resolution and interlayer adhesion determine the architectural fidelity and functional integration of the construct. The interface between printed layers, if poorly consolidated, can become a site of mechanical failure or irregular degradation. This approach provides precision in shaping the structure, allowing for customization based on the specific needs of the patient or clinical application.^69^ Nonetheless, ensuring uniformity at the microscale and maintaining biological activity of collagen during extrusion remain key challenges, calling for further refinement of printing parameters and postprocessing methods.

These diverse manufacturing techniques offer a broad range of options for the use of collagen in tissue engineering, each adapted to meet specific application requirements and desired properties. Yet, to transform these forms into clinically reliable platforms, a deeper understanding of how architectural features interact with chemical interfaces is indispensable. Only by integrating both principles—structure and interfacial design—can collagen-based biomaterials achieve reproducible performance across laboratory and clinical settings.

## 4 Collagen Composites in Tissue Engineering

Despite the various advantages of using collagen in tissue engineering, the development of biomaterials faces significant challenges. Among the notable disadvantages, the limited mechanical stability of collagen stands out, which can compromise the strength of biomaterials and their effective integration into tissues. Additionally, the rapid degradation of collagen in the biological environment can result in a limited lifespan for these materials. The complexity in obtaining specific forms and the difficulty in controlling collagen’s physical and chemical properties are also considered essential limitations.

To overcome these challenges, obtaining composites, especially incorporating inorganic materials, emerges as a promising strategy. The introduction of these species, such as carbon-based materials, oxides, or metal nanoparticles, can significantly improve the mechanical stability of collagen, strengthening the resulting biomaterials. Moreover, these inorganic composites can provide greater resistance to enzymatic degradation, extending their durability. Combining specific properties of these inorganic materials with the biological properties of collagen can also create a synergistic environment that may enhance the biological activity of these composites, especially for tissue engineering. This integrated approach represents a significant advancement in the search for more robust and effective biomaterials for clinical and therapeutic applications. The following section will list the composite materials, focusing on their physicochemical properties. The applications of these biomaterials will be explored in the next section.

### 4.1 Collagen Composites with Carbon-Based Materials

Carbon-based materials, such as carbon nanotubes (CNTs) and graphene derivatives, have gained significant attention as transformative tools in biomaterial development, particularly for tissue engineering applications. Graphene-based materials, including graphene, graphene nanoplatelets, graphene oxide (GO), and reduced graphene oxide (rGO), are distinguished by their exceptional mechanical strength, electrical conductivity, and biocompatibility.^70^ Their unique two-dimensional structure provides an extensive surface area for interactions with biological molecules, making them highly suitable for applications ranging from biosensing to tissue engineering. However, their reactivity and surface chemistry can vary substantially depending on synthesis route, oxidation degree, and residual contaminants, which may influence both cytocompatibility and degradation behavior. CNTs, on the other hand, are cylindrical nanostructures formed from rolled graphene sheets that exhibit extraordinary mechanical strength, electrical properties, and a highly customizable surface chemistry.^71^ Despite these advantages, ensuring uniform dispersion of CNTs within collagen matrices remains challenging, as agglomeration can create mechanical stress points and compromise homogeneity. These characteristics make CNTs highly versatile for applications such as drug delivery systems, where controlled release is critical, as well as in promoting cell adhesion, proliferation, and differentiation.^72^

Both graphene-based materials and CNTs bring substantial advantages to the field of tissue engineering by enhancing the mechanical properties and biofunctionality of scaffolds. Their electrical conductivity is particularly beneficial in regenerating electroactive tissues, such as cardiac and neural tissues, where electrical stimulation plays a key role in cellular alignment and maturation. Nevertheless, balancing conductivity with biocompatibility requires precise tuning; excessive charge accumulation or uncontrolled electron transfer can trigger oxidative stress and unwanted inflammatory responses. Furthermore, the inherent antimicrobial properties of these carbon-based materials reduce the risk of infection in biomedical implants. Still, the antimicrobial mechanism—often linked to oxidative membrane damage—can overlap with pathways of mammalian cell stress, underscoring the need for controlled exposure and surface modification. As a result, carbon-based materials are not only improving the structural integrity of biomaterials but are also enabling new functionalities, such as the integration of electrical stimulation and targeted drug delivery.^73,74^ Their future success will depend on reproducible functionalization methods, standardized cytotoxicity assays, and detailed understanding of how electronic and mechanical cues jointly influence cellular behavior. These advancements represent a significant step forward in the development of innovative scaffolds and implants for tissue engineering, designed to meet the complex demands of regenerative medicine, as discussed in this section and summarized in Table 1.

**Table 1:** Collagen Composites with Carbon -Based Nanoparticles for Tissue Engineering

material/composite	key application/focus	primary outcome	reference
collagen type I with graphene (0–65%)	cardiac/neural platforms	promoted alignment and maturation of cardiac cells with electrical stimulation (in vitro)	^71^
fish skin collagen with graphene (1–10%)	bone engineering	enhanced mechanical strength and showed osteogenic differentiation potential (in vitro)	^72^
collagen type I with graphene (10–60%)	neural tissue support	showed immunocompatibility and supported the growth of CNS astrocytes and microglia	^73^
collagen type I cryogel with graphene (0.1–1%)	spinal cord injury repair	improved functional recovery and reduced neuroinflammation with electrostimulation (in vivo)	^74,75^
collagen type I with graphene oxide (5–90 μg/mL)	cardiac tissue engineering	upregulated key cardiac genes and increased cell migration by 70% (in vivo)	^76^
decellularized goat pericardium with graphene oxide	antimicrobial biomaterials	exhibited good cell viability and effective antimicrobial activity (in vitro)	^77^
mineralized collagen with graphene oxide (3 mg/mL)	dental implant coatings	showed high antimicrobial activity and improved gingival fibroblast proliferation	^78^
commercial collagen with graphene oxide (1 μg/mL) coating	bone/defect healing	increased cell growth and bone formation, enhancing alveolar bone regeneration (in vivo)	^79^
collagen scaffolds with GO (0.1 wt %) and rGO	bone engineering	both supported cell spreading; rGO was more effective than GO for osteogenic differentiation (in vivo)	^81^
collagen/PLGA with graphene oxide	myogenesis induction	the electrospun scaffold induced spontaneous myogenesis and increased cell proliferation	^82^
collagen with GO (0.002 mg/mL) and sericin	angiogenesis	showed low hemolysis (<0.5%) and promoted blood vessel formation in CAM assay (in vivo)	^83^
collagen type I with GO and *N*-acetylcysteine	diabetic wound healing	improved cell proliferation, enhanced collagen gene expression, and nearly completed wound repair (in vivo)	^84^
collagen with graphene oxide and Sr^2+^	bone/vascular regeneration	facilitated osteogenic differentiation and stimulated angiogenesis (in vitro and in vivo)	^85^
collagen with MWCNTs (0.5–1.5%)	general tissue scaffolds	enhanced cellular adhesion and proliferation	^88^
collagen hydrogel with CNTs	drug delivery	provided stability to the hydrogel, enhancing the release of gentamicin sulfate	^89^
collagen with CNTs (10–100 ppm)	stem cell differentiation	increased alkaline phosphatase activity and extracellular matrix mineralization	^92–95^
collagen with CNTs (1 μM to 20 μM)	wound healing	enhanced mechanical properties (46x), cell viability (>70%), and accelerated wound healing (>70%)	^98,99^
electrospun collagen fibers with CNTs	tissue regeneration	had higher mechanical strength and electrical properties; increased type III collagen synthesis	^109^
collagen hydrogel with SWCNTs	cardiac regeneration	showed improved electrical and mechanical properties, making it promising for cardiac applications	^113^
collagen and CNT coating on PET tapes	ligament engineering	formed artificial knee ligaments with reduced hemolysis and active connective tissue growth (in vivo)	^117^

Ryan et al. developed composites by the casting method using collagen type I and graphene (0–65% w/w).^75^ he results revealed a significant increase in electrical conductivity when using 32% graphene in a hydrated state (0.6 S/m). Additionally, these films enhanced the proliferation of murine cardiac fibroblasts and embryonic stem cells (ESC-CMs). When electrical potential was applied to these films, they promoted improved alignment and maturation of the cells, demonstrating potential applications as biohybrid platforms for cardiac and neural systems. This study reinforces the importance of optimizing graphene loading, as higher fractions may lead to brittleness or decreased permeability, affecting nutrient diffusion. Collagen scaffolds derived from fish skin (Catla fish) combined with graphene (1–10%) exhibited enhanced mechanical properties, with strength increased by up to 3-fold. These scaffolds also showed cell viability above 90% with MG-63 cell lines and demonstrated osteogenic differentiation potential, confirming their ability to support osteogenesis.^76^ However, the origin of collagen and the degree of cross-linking can significantly influence these outcomes, suggesting that standardized fabrication conditions are essential for reproducibility.

Electroactive films based on type I collagen and graphene (10–60%), produced by Maughan et al., demonstrated improved electrical conductivity (1.5 S/m with 60% collagen), immunocompatibility, and the ability to support the growth and proliferation of central nervous system (CNS) astrocytes and microglia. These cells exhibited morphologies and cytokine expression profiles typical of noninflammatory resting phenotypes (Figure 6).^77^ Such findings suggest that carbon-based reinforcements can be engineered not only to transmit electrical cues but also to modulate immune responses, an underexplored yet decisive parameter for clinical translation. Further studies, such as those by Agarwal et al., investigated type I collagen-graphene (0.1–1%)-cross-linked cryogels for creating nerve conduits for in vivo spinal cord regeneration postinjury.^78,79^ These cryogels, when subjected to electrostimulation, demonstrated improved functional recovery, reduced neuroinflammation, and accelerated axonal regeneration. Still, the transition from small-animal models to large-scale validation will require long-term monitoring of electrical stability and biodegradation under physiological conditions.

**Figure 6:**
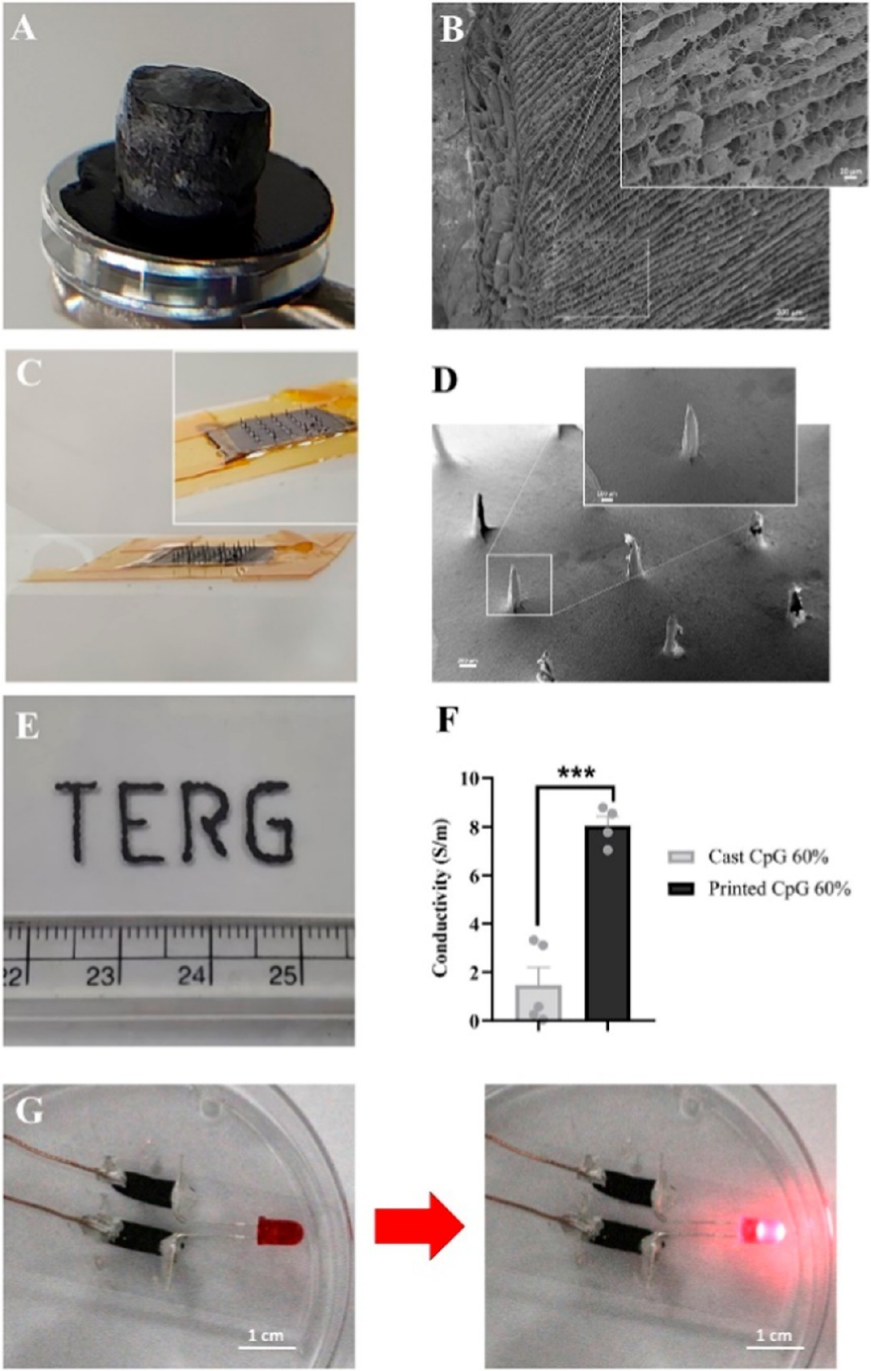
(a) CpG60% was used to fabricate lyophilized porous conductive scaffolds (b) when imaged using scanning electron microscopy (SEM) show the porous internally aligned channels. Scalebars = 200 and 20 μm (inset). (c) Microneedle arrays were fabricated using the composite to produce a 5 × 5 microneedle set. (d) Each needle was 2.5 mm high and with a tip diameter of 40–80 μm when imaged at higher power using SEM the fine bore and sharp tips can be clearly seen on the individual needles. Scalebars = 200 and 100 μm (inset). (e) The development of the CpG60% as a printable bioink allowed for the control and fine printing of complex 3D geometries (f) with excellent conductivity. (g) To demonstrate the conductive function of the CpG60% bioink, a simple circuit was printed to power an LED. “Reprinted with permission under a Creative Commons CC-BY 4.0 from.^77^ Copyright (2022) Elsevier.”

The use of GO (5–90 μg/mL) associated with collagen type I also resulted in electroactive composites, used for the adhesion of neonatal cardiomyocytes and increased expression of cardiac genes through electrostimulation. The scaffolds exhibited an electrical conductivity of 10^–4^ S/m, classifying them as semiconductors. Additionally, proliferation tests with HUVEC cells revealed that the scaffolds not only supported cell proliferation but also upregulated key genes such as TrpT-2, Cx43, and Actn4, which play vital roles in cardiac tissue function. In vivo tests in NMRI mice further demonstrated a remarkable 70% increase in cell migration.^80^ Although these results are promising, the variability in graphene oxide purity, oxidation level, and reduction state can substantially alter biocompatibility and electrical behavior, emphasizing the necessity for rigorous material characterization prior to biological assessment.

Deepak et al. developed biohybrid composites based on a biohybrid scaffold of decellularized goat pericardium (rich in collagen) and GO (6.2–2000 μg/mL), aiming to enhance their mechanical and biological properties. The GO–DCP biohybrid scaffold was fabricated using immersion coating techniques, with approximately 36% of GO successfully bound to the decellularized matrix. Moreover, treatment with the highest concentration (2000 μg/mL) resulted in a cell viability of 69.8 ± 4.0%. Antimicrobial tests revealed the absence of colony formation at concentrations above 125 μg/mL.^81^ It was observed that these biohybrid composites have the potential to be applied as biomaterials for cellular regeneration, exhibiting improved cell adhesion and antimicrobial potential. However, the trade-off between antimicrobial efficacy and cytocompatibility at higher GO concentrations highlights the importance of dose optimization and interface stabilization to avoid excessive oxidative stress.

Dental implant coatings using GO (3 mg/mL in 30% ethanol solution) with mineralized collagen showed high antimicrobial activity, significantly improving human gingival fibroblasts’ adhesion, cytoskeletal organization, and proliferation.^82^ Coatings on commercial collagen scaffolds with GO (1 μg/mL) also proved effective for cell growth and bone formation in rats, along with stimulating the healing of class II furcation defects in dogs, enhancing alveolar bone regeneration by 70%.^83^ GO composites also demonstrated effectiveness in detecting xenogeneic collagen cosignatures, providing a strategy to regulate the thermal stability of collagen.^84^ Collagen scaffolds with GO (0.1 wt %) and reduced GO (rGO) were produced and SEM analysis revealed that MC3T3-E1 osteoblast cells exhibited spreading and elongation on the scaffolds. When implanted subcutaneously on the backs of rats, revealing that rGO is more effective for osteogenic differentiation than GO.^85^

Electrospun biomimetic scaffolds composed of PLGA (200 mg/mL), collagen (30 mg/mL), and GO (10 mg/mL) were also efficient in inducing spontaneous myogenesis. Also, the adhesion and proliferation tests showed an increase, particularly in the groups with collagen and GO.^86^ Jayavardhini et al. enhanced the angiogenic activity of GO (0.002 mg/mL) scaffolds with collagen by adding small amounts of sericin (Figure 7). This study presented results from a hemolysis assay evaluating erythrocytes in contact with the scaffolds, which showed a hemolysis rate of less than 0.5%. Additionally, in the chick embryo chorioallantoic membrane (CAM) assay, blood vessel formation was observed in samples containing the scaffolds.^87^ Qian et al. created implantable scaffolds based on collagen and GO (1:5 volume ratio) for sustained release of *N*-acetylcysteine (100 mg/L, 500 mg/L and 1000 mg/L) to evaluate wound healing in diabetic rats. The in vitro proliferation analyses revealed that the group containing collagen, GO, and 500 mg/L of *N*-acetylcysteine exhibited superior results (95.44 ± 6.10%). Similarly, RT-PCR results for collagen Type I (1.81 ± 0.05-fold) and collagen Type III (1.95 ± 0.06-fold) genes also showed enhanced expression. Furthermore, the in vitro keratinocyte migration assay demonstrated improved results at the 500 mg/L concentration (58.17 ± 3.67%). For the in vivo analyses, wounds treated with 500 mg/L scaffolds were nearly completely repaired.^88^ These scaffolds had a beneficial effect in improving the wound-healing process in rats. They induced the antioxidant defense system by increasing the expression levels of antioxidant enzymes in the wound tissue. Sr^2+^-releasing scaffolds, using collagen and GO composites, effectively facilitated cell adhesion, osteogenic differentiation, and promoted the secretion of angiogenic factor secretion. Specifically, more than 78% of hADSCs cultured on Sr–GO–Col scaffolds remained viable after 1 day of incubation, with viability increasing to over 88% after 3 days. Additionally, Sr–GO–Col-stimulated hADSC-conditioned medium enhanced HUVEC recruitment, promoted tube formation, and supported angiogenesis. Furthermore, hADSCs cultured on Sr–GO–Col scaffolds exhibited significantly upregulated expression of VEGF and PDGF-BB, further stimulating endothelial tube formation in vitro.^89^ The in vivo results were consistent with the in vitro findings, showing superior bone regeneration and angiogenesis outcomes. Using other biopolymers, such as alginate, polycaprolactone, and chitosan, with collagen and GO composites also emerges as an exciting strategy to enhance the osteogenic capacity of osteoblasts.^90,91^

**Figure 7:**
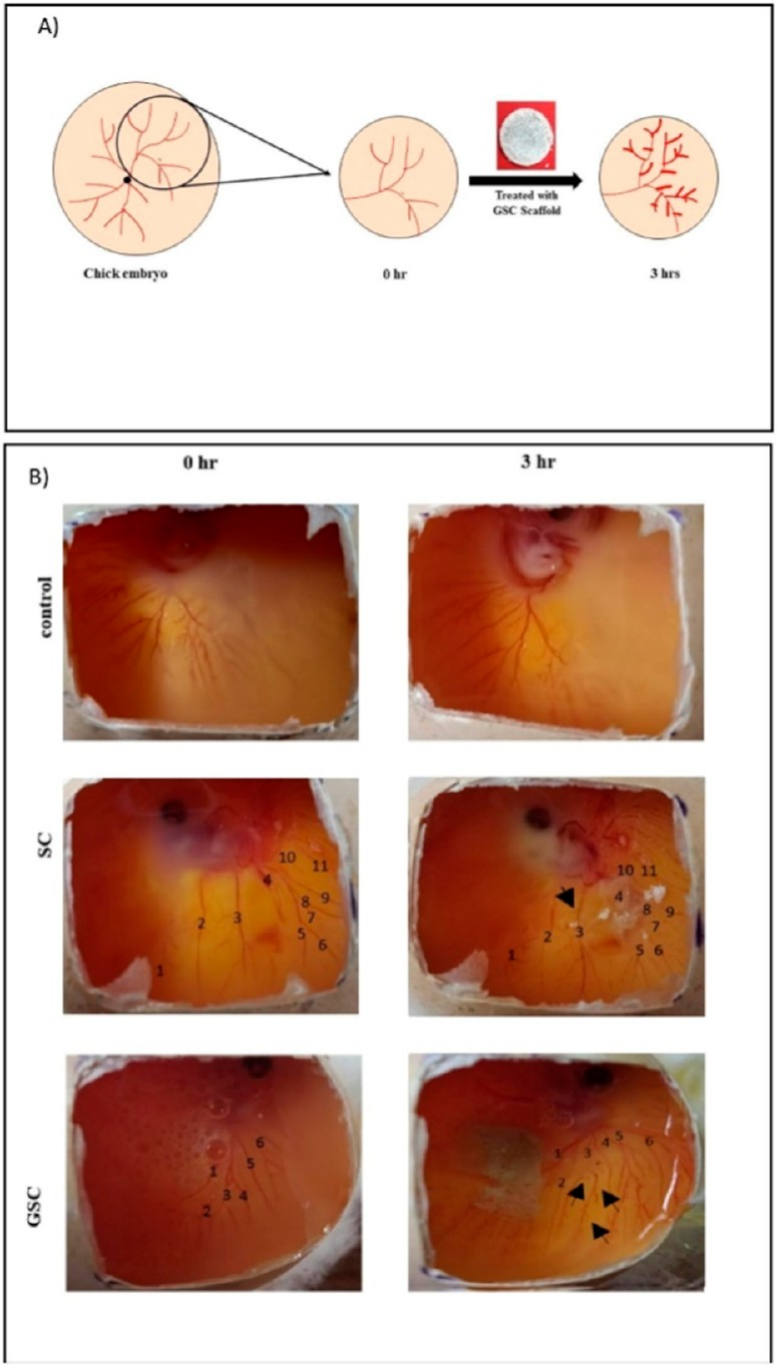
(a) Schematic representation of CAM assay performed using GSC scaffold (b) photographs taken during in vivo CAM assay in control, SC and GSC treated groups. Numbers indicate blood vessels. Black arrows indicate new blood vessels formed after incubation. “Reproduced with permission from.^87^ Copyright (2022) Elsevier.”

Another carbon-based material of interest for developing composites with collagen is CNT. These materials can exist as single-walled CNTs (SWCNTs) or multiwalled CNTs (MWCNTs) and can be functionalized with various functional groups. They also possess a high adsorption capacity, making them an attractive carrier for drug delivery systems as demonstrated by Farshidfar et al., who observed enhanced cellular adhesion and proliferation in scaffolds composed of MWCNTs (0.5–1.5%) combined with collagen (0.5%, 1%, and 1.5% w/w).^92^ Li et al. found that CNTs provide additional stability to the collagen hydrogel, enhancing the drug delivery of gentamicin sulfate.^93^ However, high concentrations of CNTs can have adverse effects, including toxicity, reduced cell migration, and inflammation in scaffolds.^94,95^ Valverde et al. analyzed type I collagen scaffolds from rat tails associated with MWCNTs at concentrations of 0.6, 1.2, 2.4, and 6.0% per unit weight of collagen I, as well as Mineral Trioxide Aggregate at concentrations of 1.6, 3.2, 6.4, and 12.8% per unit weight of collagen I. They observed that the samples showed no cytotoxicity, but the groups with MWCNTs exhibited a lower percentage of cellular migration.^94^ Hirata et al. analyzed MWCNT-coated sponges and observed no significant difference in DNA content. However, ALP activity in the cells was significantly higher on the MWCNT-coated sponges compared to the uncoated ones. Additionally, calcium and OPN contents were measured and found to be significantly greater on the MWCNT-coated sponges than on the uncoated sponges. Also, in vivo tests demonstrated successful bone tissue formation within the pores of the MWCNT-coated sponge.^95^

Collagen scaffolds with CNTs (10 or 100 ppm) significantly increase alkaline phosphatase activity and extracellular matrix mineralization due to the increased stiffness and strength of the CNTs.^96^ This provides potential for controlling the differentiation of mesenchymal stem cells using these scaffolds. Notably, cells exhibit favorable adhesion to these composites, spreading more easily than pure collagen.^97–99^ According to Kim et al., CNTs (0.1, 0.25, 0.5, and 0.7 w %) interact with collagen (1.5 mg/mL) at the molecular level, relaxing the helical collagen fibrils and leading to their elongation.^100^ This elongation, particularly of the D-period, may be responsible for rapid and efficient cellular differentiation.^101^ Vedhanayagam et al. observed that collagen (0.5 μM) scaffolds with CNTs (1 μM to 20 μM) have mechanical properties enhanced by 46 times compared to pure collagen, along with better cell viability (greater than 70%) and accelerated wound healing (more than 70%).^102^ The increased mechanical strength is due to the alignment of the 1D structures of CNTs with the collagen fibers.^103^ Additionally, Mao et al. found that the production of sulfated glycosaminoglycans increases (above 1.5 μg per μg/DNA) when using CNTs (≈50 μg/mL) with collagen (0.1 wt %).^104^ These components are essential for the extracellular matrix that fills the spaces between cells. Mao et al. fabricated CNT probes (1 mg) with collagen (0.1 wt %) to label human mesenchymal stem cells, ensuring cell labeling for up to 2 weeks (above 15%) and cell viability more than 90%.^105^ Injectable chitosan/collagen hydrogels, as well as coatings with COOH-functionalized CNTs, significantly increased the bioactivity of these materials.^106,107^

Furthermore, like graphene-based materials, CNTs have interesting electrical properties and can be used to produce bioelectronic devices.^108^ Reversible microfluidic devices were developed by Popovich et al. using collagen and MWCNTs (0.01 wt %), suitable for preliminary tests of the resistance and thrombogenicity of various coatings and membranes.^109^ Collagen and CNT-based composite materials are promising for applications as cardiac/neural constructs, as different types of stimulus propagation patterns are required for regenerating cells (Figure 8).^110–112^ The nature of reinforcement affects the electrical conductivity of the scaffold and also determines the type of cell it can support for regeneration. Chi et al. produced electrospun collagen fibers with CNTs, which exhibited higher mechanical strength and improved electrical properties.^113^ When these fibers were in contact with fibroblasts, increased synthesis of type III collagen was observed. Incorporating CNTs into collagen hydrogel can promote nerve growth factor and brain-derived neurotrophic factor, providing excellent 3D conditions for the growth of mesenchymal stem cells.^114^ The combination of GO and CNTs can also be an interesting alternative for collagen-based scaffolds, as they can mutually induce collagen molecules to self-organize and form aligned fibril microstructures.^115^

**Figure 8:**
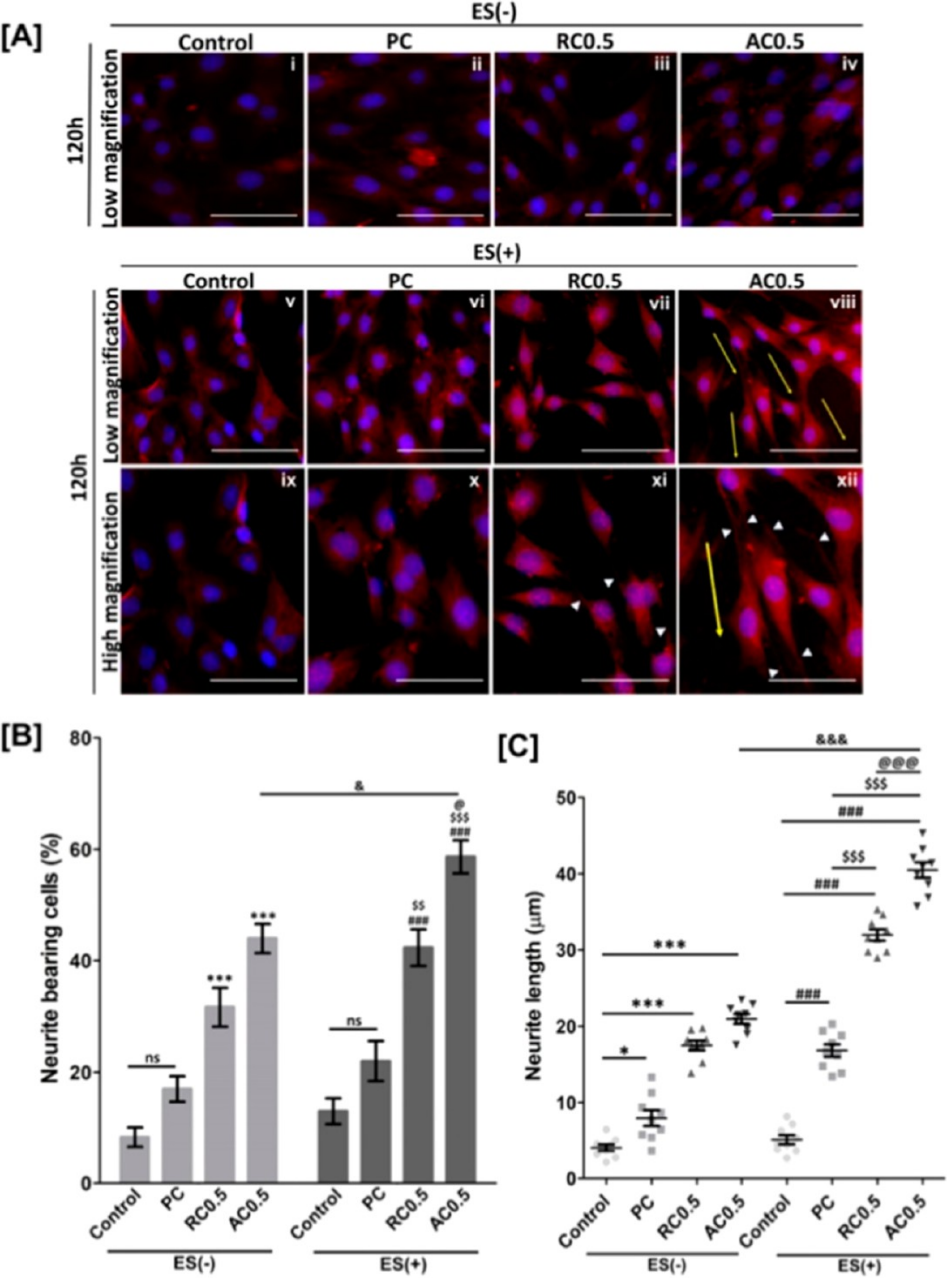
(a) Immunofluorescence study of HT-22 cells with MAP2 antibody to check the morphology of the cells cultured on different types of PC-MWCNT scaffolds before (i–iv) (scale bar: 50 μm) and after (v–xii) electrical stimulation. (Upper panel scale bar: 50 μm; Lower panel scale bar: 30 μm). Yellow arrows indicated the alignment direction. White arrow-heads indicated the neurites. (b) Number of neurite bearing cells (* denotes *p* < 0.05 vs control of ES(−) condition; # denotes *p* < 0.05 vs control of ES­(+) condition; $ denotes *p* < 0.05 vs PC of ES­(+) condition; @ denotes *p* < 0.05 vs RC0.5 of ES­(+) condition; and denotes *p* < 0.05 vs AC0.5 of ES(−) condition). (c) Length of neurites on PC and different types of PC-MWCNT scaffolds w/o and with electrical stimulation for 5 days. “Reproduced with permission from.^110^ Copyright (2022) Elsevier.”.

Electrical stimulation of these composites can also be helpful for the fine control of substances in collagen-based hydrogels, such as nerve growth factor.^116^ Sun et al. integrated SWCNTs into collagen hydrogels, creating a two-dimensional microscopic environment essential for the growth and function of cardiomyocytes, with improved electrical and mechanical properties, making these materials promising for cardiac regeneration.^117^ Cells like cardiomyocytes and human dermal fibroblasts can also be integrated into these composites, enhancing the cell functions of gels with improved electrical conductivity.^118–120^ Gerasimenko et al. coated PET tapes with collagen and CNT coatings to form artificial knee ligaments, showing reduced hemolysis of the coated material, along with more active growth of connective tissue with mature collagen fibers at the implantation site.^121^ These coatings were also applied to titanium, showing increased cell proliferation.^122^ For collagen composites, doping CNTs with boron can improve the electrical properties of these materials.^123^

Carbon-based nanomaterials have clearly redefined the design of collagen composites, offering a unique combination of mechanical reinforcement, conductivity, and biological activity that extends far beyond traditional fillers.^124^ Graphene derivatives and CNTs have shown the ability to strengthen collagen scaffolds, facilitate electroactive tissue regeneration, and introduce antimicrobial or drug-release functions. Yet, their effectiveness depends critically on how they are dispersed, integrated, and stabilized within the biopolymer matrix. Subtle variations in oxidation state, surface chemistry, or nanotube alignment can drastically alter conductivity, biocompatibility, and degradation, making reproducibility one of the major bottlenecks in this field. Moreover, while electrical stimulation and nanoscale rigidity enhance cell differentiation, uncontrolled charge transfer, local heating, or excessive stiffness may compromise long-term biocompatibility and tissue remodeling. The biological impact of functionalization routes and dopants—such as boron or oxygen-containing groups—remains insufficiently understood, as do the long-term fate and clearance of nanocarbon residues in vivo. Moving forward, progress will rely less on empirical optimization and more on mechanistic insight into how architecture, interface chemistry, and electronic properties collectively shape cellular behavior. Establishing standardized synthesis and evaluation protocols, coupled with predictive modeling and long-term biological assessment, will be essential to transform these carbon–collagen systems from experimental constructs into reproducible, safe, and clinically viable materials for regenerative medicine.

### 4.2 Collagen Composites with Metal Nanoparticles

Metallic materials, especially metallic nanoparticles, have proven to be key components in the advancement of biomaterials, offering distinct advantages for tissue engineering applications. Nanoparticles of metals like silver (Ag), gold (Au), copper (Cu), zinc (Zn), and cobalt (Co) stand out for their unique properties derived from their small size and high surface area, making them highly effective in various biomedical applications.^125^ Ag nanoparticles, well-known for their potent antimicrobial properties, have been extensively used in wound healing and infection prevention, making them valuable in medical treatments.^126^ Au nanoparticles, with their excellent biocompatibility and tunable optical properties, hold significant potential for imaging and diagnostic applications, enabling noninvasive medical diagnostics.^127^ Cu nanoparticles are celebrated for their biocompatibility and ability to promote tissue regeneration, particularly through their angiogenic properties.^128^ Zn nanoparticles contribute to bone regeneration and wound healing by enhancing mineralization and reducing inflammation,^129^ while Co nanoparticles stimulate angiogenesis by mimicking hypoxia-inducing factors, supporting vascular and connective tissue formation.^130^ Despite the inherent compatibility of collagen with the extracellular matrix, its pure form lacks mechanical strength, limiting its durability in long-term tissue engineering applications.^131^ To address this challenge, integrating metallic nanoparticles with collagen has been explored as a strategy to enhance the protein’s stability and mechanical integrity. This combination not only improves the structural properties of the scaffolds but also leverages the bioactive and antimicrobial benefits of the nanoparticles, paving the way for more robust and functional biomaterials tailored for regenerative medicine. The studies involving the development of collagen composites with metallic nanoparticles will be discussed in this section, and the results are summarized in Table 2.

**Table 2:** Collagen Composites with Metal Nanoparticles for Tissue Engineering

material/composite	key application/focus	primary outcome	reference
collagen with Ag nanoparticles	antimicrobial skin substitute	was effective against various pathogens, reduced inflammation, and supported skin cell proliferation (in vivo)	^128^
collagen with Ag nanoparticles and histidine	burned/infected skin repair	maintained bactericidal properties and improved mechanical strength, promoting tissue regeneration	^129^
collagen with Ag nanoparticles	peripheral nerve repair	adsorbed laminin and promoted nerve regeneration with improved nerve conduction velocity (in vivo)	^130^
collagen encapsulating Ag nanoparticles	bone fracture healing	resulted in early fracture gap closure by promoting mesenchymal stem cell activity (in vivo)	^131^
collagen with Ag nanoparticles and BMP-2	infected bone defects	provided a dual-action approach with antimicrobial (Ag^+^) and osteoinductive (BMP-2) effects	^132^
collagen with Au nanoparticles	biosensing	developed as a colorimetric sensor for the selective detection of glucose and heparin	^133^
collagen with Au nanoparticles	bone tissue regeneration	promoted osteogenic differentiation, mineralization, and advanced bone regeneration (in vitro and in vivo)	^134^
Au-coated collagen nanofibers	stem cell differentiation	it was biocompatible and enhanced myocardial and neuronal differentiation of mesenchymal cells	^135^
collagen with Cu and bioglass	osteomyelitis treatment	demonstrated strong antimicrobial activity and significant improvements in osteogenesis and angiogenesis	^137^
collagen with Cu nanoparticles	vascularization/angiogenesis	significantly increased microvessel formation and improved the viability of skin flaps	^138^
collagen with Ca/Zn coatings	orthopedic implants	allowed for controlled release of Zn^2+^, enhancing both osteogenic and angiogenic properties	^143^
collagen with Zn nanoparticles and bioglass	skin regeneration	improved conditions for wound closure with antibacterial properties and minimal scarring	^145^
collagen type I with Co nanoparticles	extracellular matrix assembly	affected collagen assembly, resulting in greater heterogeneity and enhanced mechanical properties	^146^
collagen/alginate with Co^2+^ and BMP-2	bone tissue regeneration	stimulated angiogenesis and osteogenesis through controlled ion release, increasing bone formation	^147^

In light of increasing antimicrobial resistance, Ag is gaining prominence in developing biomaterials, especially for cross-linking collagen scaffolds. Although Ag is effective against bacteria, it is also toxic to mammalian cells. A promising alternative is the use of Ag nanoparticles instead of ionic Ag (Ag^+^). When these nanoparticles are coated with biomolecules such as collagen, they retain their bactericidal and bacteriostatic properties but with significantly reduced cytotoxic effects. Consequently, collagen-coated Ag nanoparticles are becoming increasingly popular in developing bioactive materials with anti-infective properties for tissue engineering applications. Alarcon et al. conducted tests on the microbial activity of collagen-coated Ag nanoparticles.^132^ These scaffolds demonstrated effectiveness against pathogens such as *S. aureus*, *S. epidermidis*, *E. coli*, and *P. aeruginosa* even at low concentrations, highlighting their strong anti-infective capabilities. When used as skin implants, these scaffolds were shown to reduce the expression levels of the pro-inflammatory cytokine IL-6, while simultaneously supporting the proliferation of primary skin cells, thereby acting as effective skin substitutes.

**Table 3:** Collagen Composites with Oxide-based Nanoparticles for Tissue Engineering

material/composite	key application/focus	primary outcome	reference
collagen with Porous HA/β-TCP	bone regeneration	the component ratio was found to affect scaffold porosity and in vivo behavior	^165^
collagen with HA (Robocasting)	bone defect repair	promoted bone marrow stromal cell proliferation and enhanced cell repair (in vitro and in vivo)	^166^
collagen with HA mesoporous microspheres	drug delivery/bone repair	showed a high cell proliferation rate and was identified as a candidate for drug delivery systems	^169^
collagen (30%) with HA (70%)	bone regeneration	was biocompatible and increased bone recovery by 3.4 times over 8 weeks (in vivo)	^171^
collagen with Zn-doped HA nanoparticles	bone tissue regeneration	demonstrated high biocompatibility, tissue integration, and good degradation potential (in vivo)	^175^
PLGA/Collagen with HA (Electrospun)	osteogenic differentiation	the multilayered scaffold controlled the adhesion, proliferation, and differentiation of osteoblast-like cells	^178^
collagen/Sodium alginate with TiO_2_ nanoparticles	periodontitis treatment	supported and stimulated osteogenesis through a Runx2 signaling mechanism	^187^
collagen with PVP-coated TiO_2_ nanoparticles	wound healing/skin regeneration	resulted in a biomaterial with higher tensile strength, making it an effective skin regenerator	^188^
g-PMMA-Collagen with PdO–TiO_2_ nanocomposites	bone tissue engineering	exhibited greater mechanical strength and enhanced osteogenic activity without toxicity (in vitro)	^189^
collagen cross-linked with various metal oxides	skin regeneration	A comparative study found ZnO to be the most effective for skin regeneration due to superior mechanical, viable, and antimicrobial properties	^190^
collagen with ZnO nanoparticles and essential oil	burn dressings	the material was biocompatible, antimicrobial, and accelerated wound healing	^193^
collagen with bioactive glass (BG)	bone tissue engineering	increased the scaffold’s compressive strength and allowed for controlled drug delivery	^194^
collagen with bioactive glass (BG) fibers	bone defect treatment	enhanced cell adhesion and proliferation; significantly increased osteogenic gene expression levels	^196^
collagen with amine-coated Fe_2_O_3_ nanoparticles	bioimaging/implants	the material was nontoxic and had paramagnetic properties, enabling its use as an MRI probe	^186^
collagen with USPIO (Fe_2_O_3_) nanoparticles	implant tracking (MRI)	the scaffold was highly biocompatible and allowed for noninvasive visualization of the implant’s location and function	^198^

Song et al. reported the development of a scaffold for skin tissue, comprising collagen, Ag nanoparticles, and histidine.^133^ The scaffold maintained its bactericidal properties, and the inclusion of histidine improved the mechanical strength (0.137 and 0.157 MPa) of the biomaterial. This enhanced strength facilitated the scaffold’s application to burned and infected skin, promoting effective tissue regeneration within 3 weeks. Further exploring the advantages of incorporating Ag nanoparticles into collagen, Ding et al. highlighted benefits beyond antimicrobial properties.^134^ Their research involved a collagen-Ag nanoparticles scaffold with homogeneous distribution, demonstrating the ability to adsorb laminin and promote the repair and regeneration of damaged peripheral nerves in animal models. The study showed successful sciatic nerve regeneration, and when comparing scaffolds with and without Ag, it was noted that the presence of Ag nanoparticles led to greater laminin adsorption, thicker myelin sheath regeneration (1.2 ± 0.5 mm), improved nerve conduction velocity, and increased nerve potential amplitude (0.70 ± 0.44 mV) compared to scaffolds made of collagen alone.

The use of biomaterials in bone fracture healing through the proliferation and osteogenesis of mesenchymal stem cells is well-established. Zhang et al. used Ag nanoparticles encapsulated in collagen to form a fracture callus in a mouse femoral fracture model, which resulted in the early closure of the fracture gap.^135^ The connection between the callus and the fractured bone ends was facilitated by multiple mechanisms like chemotaxis of mesenchymal stem cells and fibroblasts to the fracture site, induction of mesenchymal stem cell proliferation, and promotion of osteogenic differentiation of mesenchymal stem cells through the activation of TGF-β/BMP signaling pathways (Figure 9). Thus, scaffolds composed of Ag nanoparticles and collagen present a viable alternative for bone fracture healing. In the same context, Sun et al. developed a scaffold made of collagen and Ag nanoparticles with bone morphogenetic protein 2 (BMP-2).^136^ This scaffold offers a novel approach for treating contaminated or infected bone defects by releasing Ag^+^ ions for their antimicrobial properties while leveraging BMP-2 for osteoinductive effects, thereby promoting bone regeneration in infected wounds. Moreover, particle size and morphology, particularly in the case of Ag nanoparticles, play a decisive role in biological performance and safety profiles.^137–139^ Previous studies discussed herein indicate that the biological performance of Ag-based collagen composites is strongly governed by particle size and morphology, with reported systems employing predominantly spherical Ag nanoparticles spanning a broad size range (∼1–100 nm). This wide dispersion in structural parameters hampers direct comparison across studies and underscores the need for standardized evaluation frameworks that explicitly correlate nanoparticle size and morphology with biological and functional outcomes.

**Figure 9:**
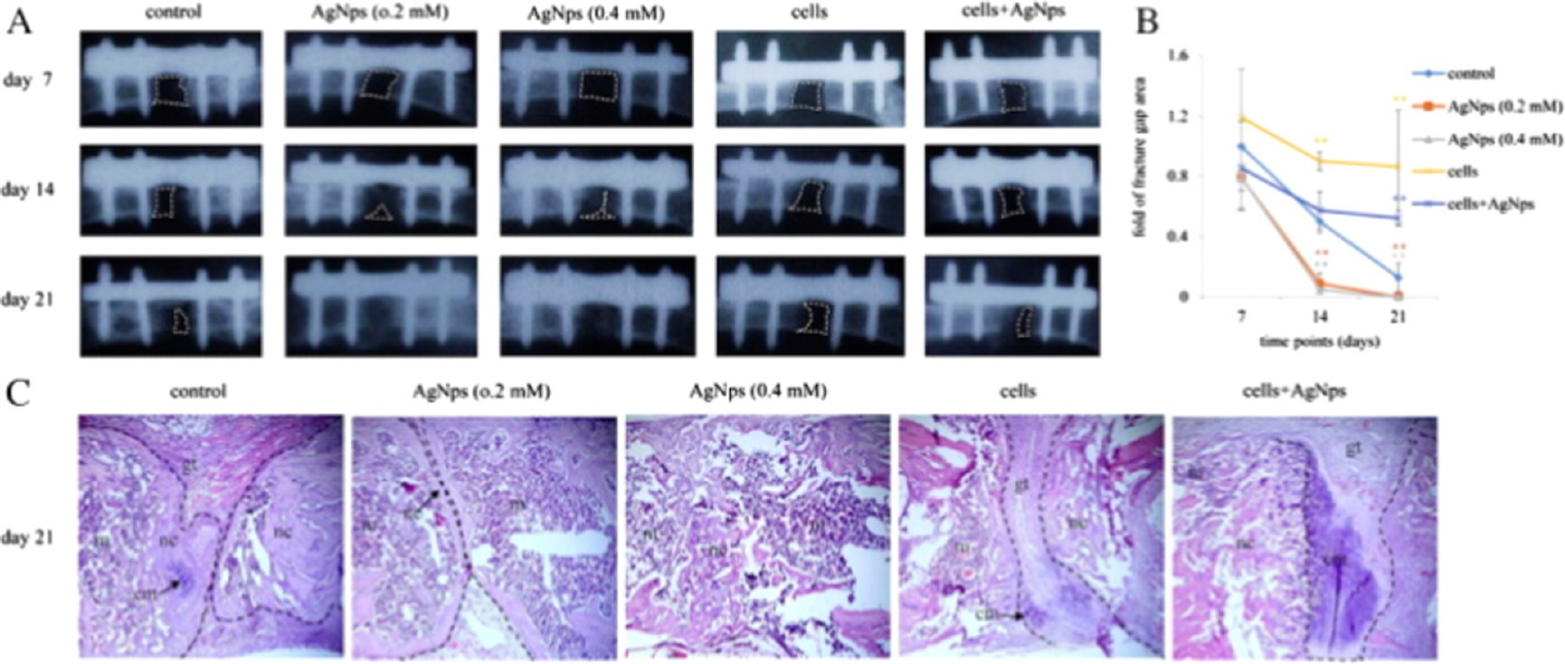
Silver nanoparticles promote fracture healing in mice. (a) Plain X-ray radiograph of the fracture sites was taken at days 7, 14, and 21 after operation, broken line demarcates the unfilled fracture gap. (b) The areas of the facture gap of each treatment groups at different postoperative days were quantified and shown. The area of the fracture gap of each group was calculated. *y*-axis indicated the fold of fracture gap area, which was calculated relative to the fracture gap area of the control group at day 7. (c) Mice were sacrificed on postoperative day 21, fractures sites were processed for histology. Hematoxylin and eosin staining of the middle section of the fracture site of each treatment group was shown. Broken lines indicate the two ends of the fracture femoral bone. “Reproduced with permission from.^135^ Copyright (2019) Elsevier.”

The use of Au nanoparticles has gained prominence in various applications, such as imaging, tissue engineering, and biosensors, due to their surface plasmon resonance, fluorescence, and functionalized surfaces. Unser et al. developed a sensor combining collagen and Au nanoparticles for the selective detection of glucose and heparin, utilizing a simple and versatile colorimetric process with excellent biological compatibility.^140^ This sensor shows a quantitative measurement of glucose in 50% mouse serum. However, the application of Au nanoparticle scaffolds extends beyond diagnostics. Heo et al. reported positive effects of collagen and Au nanoparticles scaffolds on the osteogenic differentiation of mesenchymal stem cells and osteoblast-like MC3T3-E1 cells.^141^ This novel approach to bone tissue regeneration significantly influenced new bone formation. The in vitro results demonstrated high cell proliferation and mineralization values, as well as the expression of Bone Sialoprotein (BSP), Osteocalcin (OCN), Type I Collagen (COL1), and Runt-related Transcription Factor 2 (Runx2) genes, which are essential in the bone formation process. Additionally, the in vivo results showed advanced bone regeneration within 8 weeks.^141^ Orza et al. studied Au-coated collagen nanofibers for growing and differentiating adult stem cells.^142^ These scaffolds were biocompatible and enhanced myocardial and neuronal differentiation processes of mesenchymal cells derived from the chorionic placental component. Meanwhile, Grant et al. examined the same material and observed increased longevity and stability of scaffolds used in soft tissue replacement.^143^

In developing scaffolds that combine bioglass, metals, and collagen, Ryan et al. proposed using Cu for treating osteomyelitis through the controlled local release of antibacterial agents from bioglass and Cu ions, alongside collagen-based regenerative processes.^144^ These scaffolds demonstrated both strong antimicrobial activity and significant improvements in osteogenesis and angiogenesis, positioning them as viable bone grafts (Figure 10). Cu ions are particularly beneficial in tissue engineering due to their ability to stimulate endothelial cells, promoting angiogenesis. Gérard et al., in their pursuit of vascularization alternatives for biomaterials, presented collagen and Cu nanoparticles scaffolds that significantly increased microvessel formation.^145^ This was attributed to the synergistic effect between Cu and various vascular growth factors, which stimulated angiogenesis preimplantation, thereby supporting the maintenance of cells within tissue-engineered devices. The study also evaluated the influence of Cu ions on enhancing the viability of skin flaps and the recovery of ischemic tissues. The pro-angiogenic effects of copper have prompted further research into its applications in tissue engineering. As reported by Marelli et al., adding Cu nanoparticles (560 ng for each sample) to collagen scaffolds increased the gels’ resistance to collagenase, improved mechanical properties, and raised the denaturation temperature, making these scaffolds more robust for biomedical applications^146^

**Figure 10:**
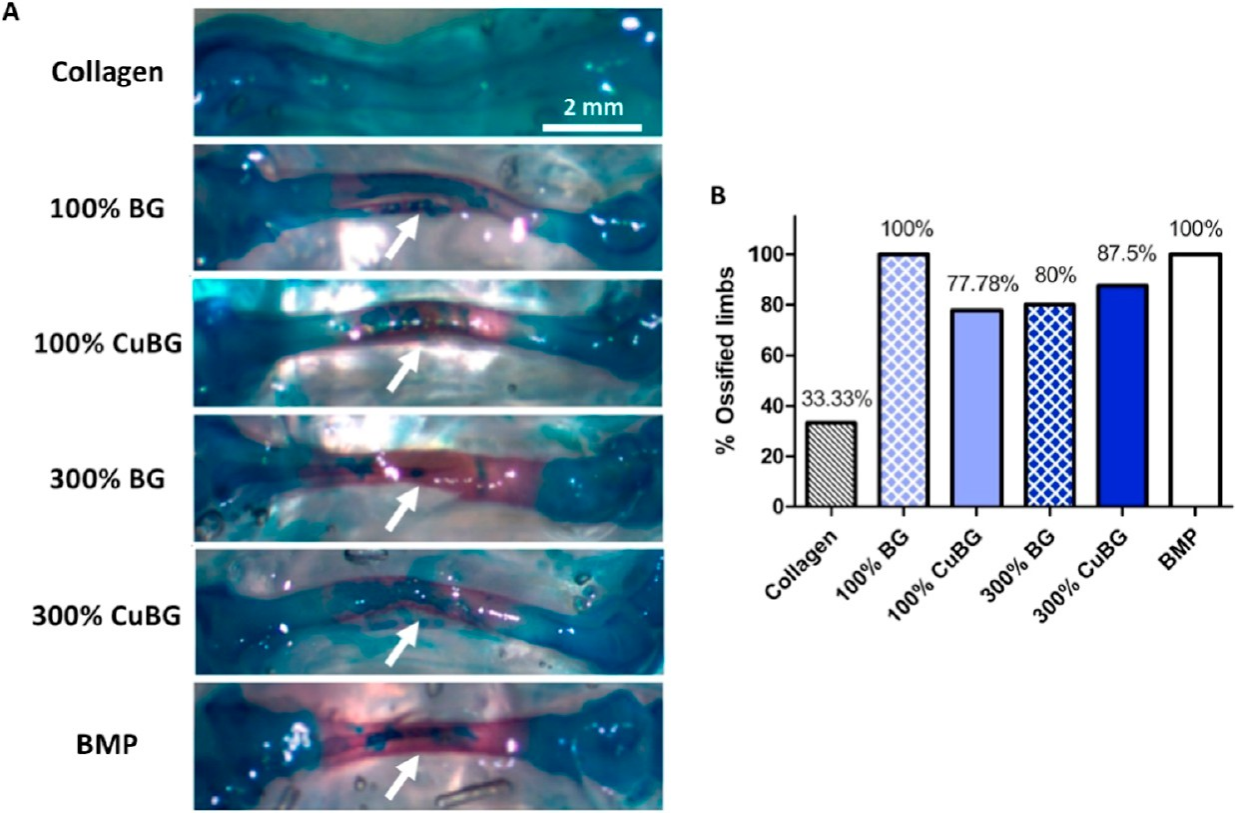
Effect of bioactive glass scaffolds on osteogenesis in a chick embryo ex ovo model. (a) Representative images of chick embryo femora at day 12 of development stained with alcian blue and alizarin red after treatments with bioactive glass scaffolds or collagen only and BMP containing scaffolds as controls (10× magnification). Note: white arrows indicate alizarin red staining. (b) Quantified percentage of harvested limbs that stained positive for alizarin red, or mineralization. “Reproduced with permission from.^141^ Copyright (2019) Elsevier.”

For applications in bone tissue engineering, collagen is favored due to its structural and compositional similarity to natural bone tissue, which consists of a calcified bone matrix, cells, and bioactive factors.^147^ Additionally, ions present in body fluids play a crucial role in bone formation, and different metals are essential for cellular functions.^148,149^ In this context, various scaffolds have been developed for bone regeneration, including those utilizing zinc (Zn) nanoparticles, which offers attractive degradation rates and mechanical properties. Qian et al. demonstrated that Ca/Zn coatings on a collagen surface allow for controlled release of Zn^2+^, thereby enhancing the osteogenic and angiogenic properties of the scaffold.^150^ This makes the scaffold highly suitable for orthopedic implants, with a significant potential for bone healing and regeneration. Moreover, Tiffany et al. developed a porous biomaterial consisting of Zn nanoparticles and collagen, which exhibited changes in the mineral phase and elastic modulus of the scaffold (Figure 11).^151^ The addition of Zn stimulates the growth and pro-osteogenic capacity of stem cells, leading to improved osteogenesis in bone lesions. These scaffolds, through the osteoinduction of the implant and enhanced osteogenic activity, serve as potential regenerative solutions for craniofacial bone defects. Zn and collagen nanoparticles are gaining significant attention in tissue engineering, where collagen serves as an excellent substrate for cell adhesion, and metal ions exert a beneficial influence. Kamrani et al. explored the combination of bioglass, Zn nanoparticles, and collagen for skin regeneration.^152^ Bioglass is known for accelerating dermal tissue regeneration by increasing cell adhesion, migration, and proliferation, while Zn^2+^ ions enhance these effects and impart antibacterial properties to the material. Consequently, scaffolds composed of Zn, bioglass, and collagen improve physiological conditions for wound closure, with the metal ensuring minimal residual scarring and improved biocompatibility of dermal implants.

**Figure 11:**
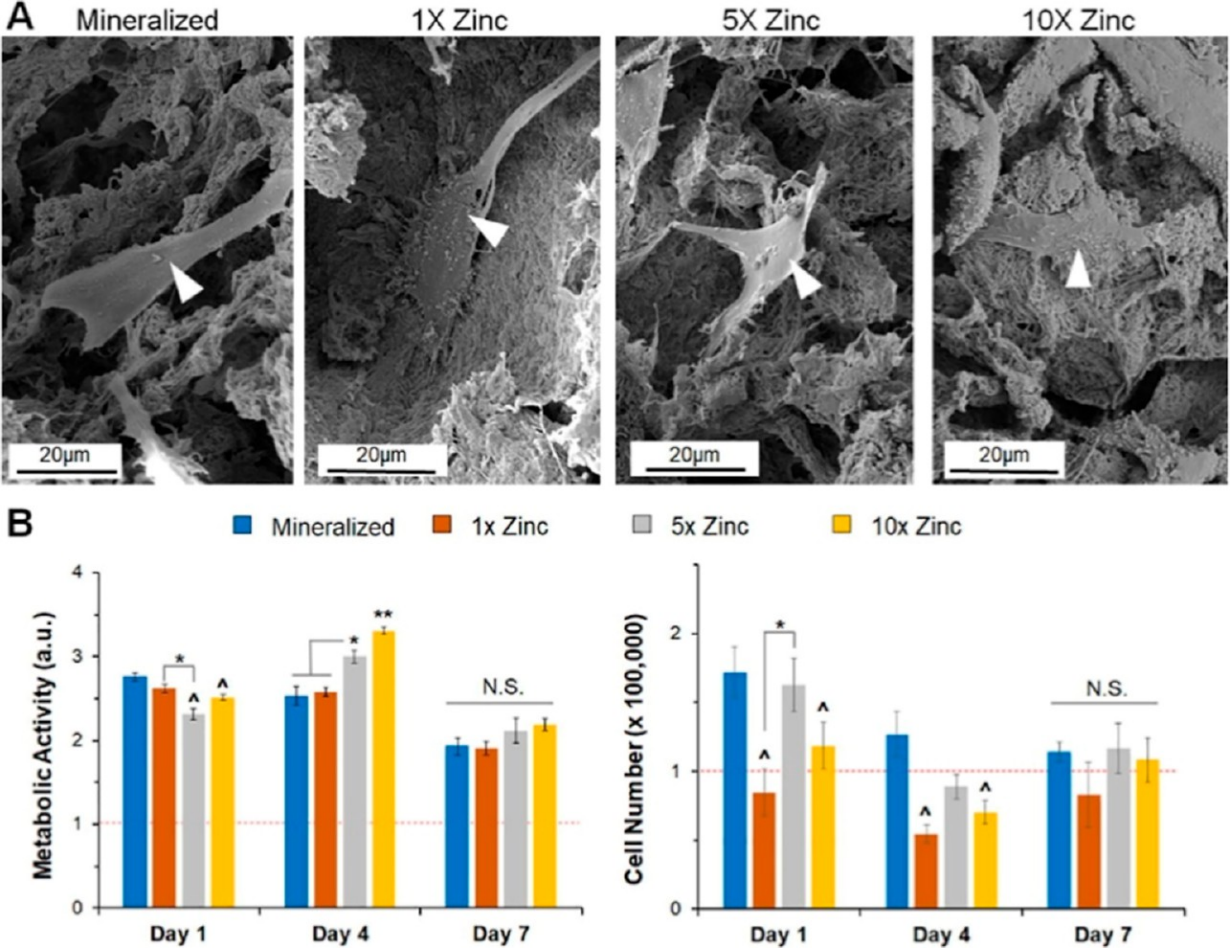
Short-term cell morphology and activity on mineralized and zinc scaffolds. (a) SEM images of seeded scaffolds show healthy cell spreading and morphology on all variants. (b) Metabolic activity for porcine derived adipose stem cells on all scaffold variants. No significant differences were seen between mineralized and 1× zinc groups for any time point. (c) Cell number showed no significance impact of zinc concentration on cell number by Day 7. “Reproduced with permission from.^148^ Copyright (2019) Elsevier.”

Given the wide range of metals and their influences on tissue engineering scaffolds, cobalt nanoparticles (Co) and collagen scaffolds are particularly notable in arthroplasty processes. McCarthy et al. highlighted that Co significantly affects the assembly of type I collagen, the primary component of the extracellular matrix.^153^ The influence of Co on collagen density results in more significant heterogeneity, thereby enhancing the mechanical properties of the scaffold. Perez et al. investigated the use of Co^2+^ in collagen/alginate/BMP-2 scaffolds.^154^ The controlled release of metal ions from these scaffolds stimulated angiogenesis and osteogenesis, leading to increased bone formation, as evidenced by new bone volume and density. This makes the material a promising option for bone tissue regeneration.

Metallic nanoparticles have emerged as highly versatile modifiers of collagen scaffolds, enabling the integration of antimicrobial, osteogenic, and angiogenic functionalities within a single material platform. Yet, their performance is governed by complex interplays between particle chemistry, dispersion, and ion dynamics within the collagen matrix.^155–157^ While Ag and Cu systems demonstrate strong antibacterial and regenerative effects, their narrow therapeutic window often challenges cytocompatibility and long-term stability. Au and Zn nanoparticles, though more biocompatible, exhibit limited mechanical reinforcement and require precise surface engineering to achieve sustained bioactivity. Co, with its hypoxia-mimetic and pro-angiogenic effects, illustrates how trace ion release can stimulate vascularization but also underscores the risks of uncontrolled ion accumulation and oxidative stress. Across these systems, the integration of metallic phases enhances collagen structural and functional performance, yet reproducibility remains hindered by variations in particle size, oxidation state, and collagen source. Developing standardized synthesis routes, long-term cytotoxicity and degradation assessments, and predictive models of ion release will be essential steps toward transforming these hybrid materials into reproducible, safe, and clinically translatable scaffolds for regenerative medicine.

### 4.3 Collagen Composites with Oxides

Oxide-based materials, such as titanium dioxide (TiO_2_), silica (SiO_2_), hydroxyapatite (HA), and zinc oxide (ZnO), have emerged as essential components in tissue engineering, offering unique properties that enhance biomaterial performance.^158,159^ These nanoparticles combine biocompatibility, stability, and functional versatility, making them ideal for supporting tissue growth and regeneration. Their ability to mimic natural tissue composition, release bioactive ions, and generate reactive oxygen species (ROS) contributes to creating antimicrobial environments that promote healing and reduce infection risks.^160–162^ Materials like ZnO and TiO_2_ are particularly noted for their antimicrobial and photoreactive properties, while HA closely resembles the mineral structure of bone, making it highly effective for bone regeneration. Additionally, oxide nanoparticles improve the mechanical properties of biomaterials, such as strength and elasticity, enhancing their ability to support tissue growth. Beyond their structural benefits, oxides like magnetite (Fe_3_O_4_) and magnesium oxide (MgO) introduce magnetic or thermal properties, enabling advanced functionalities such as targeted molecule delivery or hyperthermia treatments. When incorporated into collagen-based biomaterials, these oxide nanoparticles further enhance bioactivity and mechanical integrity, complementing the structural properties of collagen while modulating cellular behavior. This synergistic integration holds significant promise for advancing regenerative medicine, paving the way for innovative scaffolds and implants designed to meet the complex demands of tissue engineering (Table 3).

HA is likely one of the most widely used inorganic materials for cellular regeneration due to its similarities with bone constituents, low toxicity, bioabsorption, and enhanced biological properties.^163^ Collagen and HA have been used as bone-filling materials and are known to serve as osteoconductive scaffolds for bone regeneration.^164–166^ The combination of these two materials can improve both the biological and physicochemical properties of these composites.^167–171^ Porous HA/β-tricalcium phosphate (β-TCP)/collagen ceramic scaffolds were obtained by Maté-Sánchez de Val et al., demonstrating that the ratio 40/30/30 improved the bone-to-implant contact, while 60/20/20 increased the bone formation in the pores and periphery graft, thereby affecting their in vivo behavior.^172^ Lin et al. utilized the low-temperature robocasting method to fabricate 3D scaffolds made of HA and collagen with 600 μm diameter, which demonstrated a moderate mechanical strength and degradation rate, that promoted bone marrow stromal cell proliferation and improved osteogenic outcomes in vitro, as well as enhanced cell repair in vivo using a rabbit femoral condyle defect model.^173^ Human osteoblasts were seeded onto different ratios (1:99, 25:75, 50:50, and 75:25) HA/collagen scaffolds, revealing that all of the scaffolds supported human primary osteoblasts (hOBs) cell proliferation and viability.^174^

The directional control of the pores in these material-based scaffolds through controlled freezing and subsequent freeze-drying can also aid cell proliferation.^175^ Cholas et al. synthesized HA mesoporous microspheres via spray drying and incorporated them into type I collagen scaffolds with ordered interconnected microporosity via freeze-drying, resulting in a high cell proliferation rate of human osteosarcoma cell line (MG-63) and making them a candidate for drug delivery systems.^176^ Also, a microporous (1.6–1.9 μm) scaffolds (5–6 mm diameter) synthesized with HA and collagen was particularly useful for treating diseases like osteomyelitis by encapsulating therapeutic agents such as ciprofloxacin and gentamicin.^177^ Bone-mimetic composites composed of 30% collagen and 70% HA, resembling natural bone composition, have proven to be biologically active and biocompatible, providing sufficient growth support for bone marrow-derived mesenchymal stem cells (BMSCs) while promoting substance exchange (Figure 12).^178^ This approach increased bone recovery by 3.4 times in in *vivo* tests over 8 weeks.

**Figure 12:**
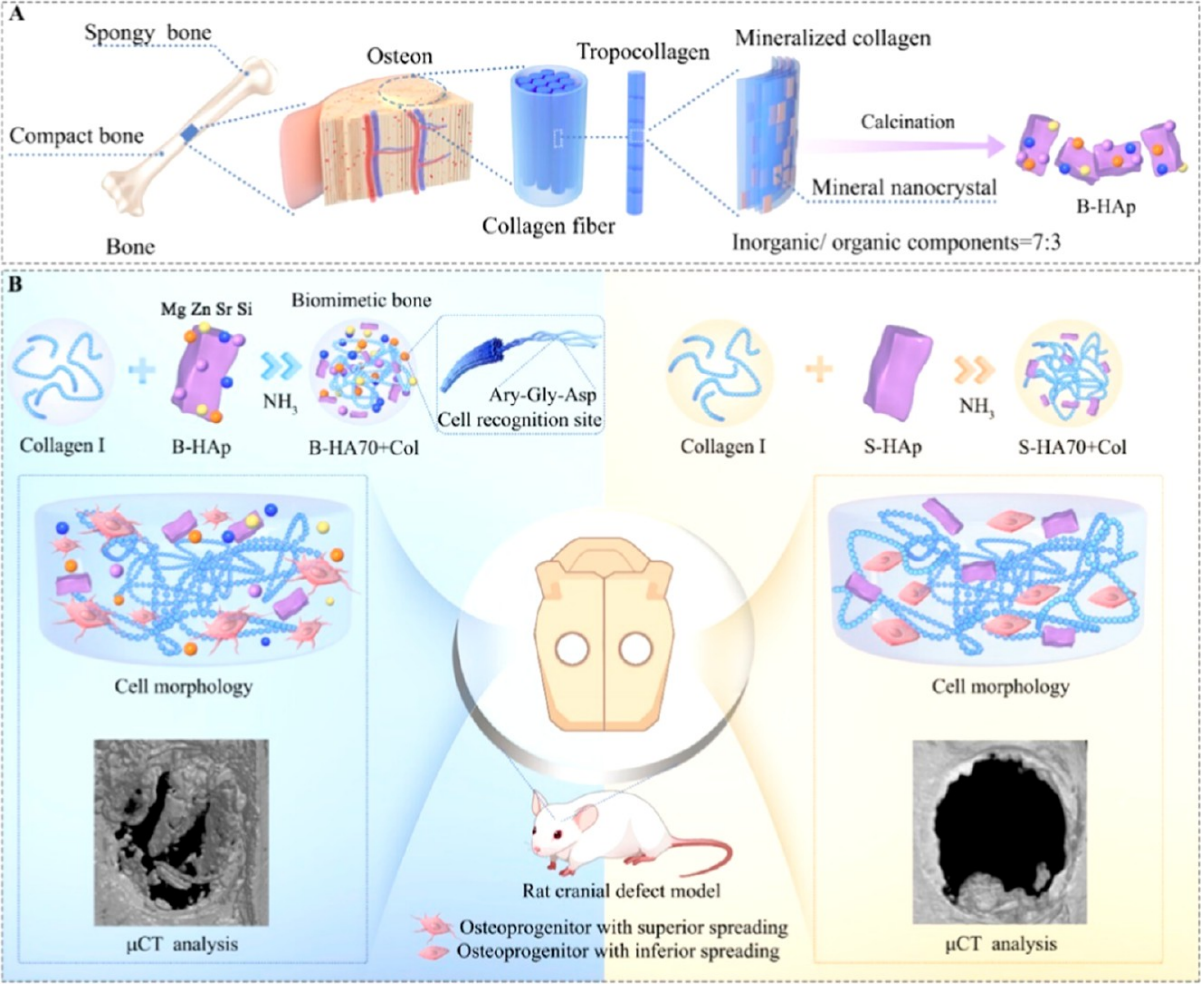
(a) Generation of B-HAp from calcination of rat bones. (b) BBCHS was produced by mixing collagen with either B-HAp or S-HAp at a ratio of 3:7, resulting in the formation of B-HA70 + Col or S-HA70 + Col, respectively. Figure 8. “Reproduced from.^175^ Copyright (2024) American Chemical Society.”

These composites are pH-sensitive and have the potential to be resorbed by osteoclast-like cells. Moreover, they enhanced bone formation on the surface by inducing TRAP-positive multinucleated cells.^179^ Collagen and HA scaffolds also show promise for administering low dose of rhBMP-2 (5 μg), inducing bridging, denser and stiffer new bone formation in vivo femoral defect after 14 days (Figure 13).^180^ 3D collagen and HA scaffolds cross-linked with 1,2,7,8-diepoxyoctane were obtained using the freeze-drying method, showing superior mechanical and biological properties for bone tissue regeneration in vivo after 12 days of radius defect in rabbits, and in vitro in MC3T3-E1 after 5 days, with a cell growth of 117.1% ± 2.9%.^181^ Wu et al. developed collagen scaffolds cross-linked with glutaraldehyde-alendronate, encapsulating Zn-doped 1% and 2% of HA nanoparticles, which demonstrated high biocompatibility, tissue integration capability, and degradation potential in an in vivo model over 6 weeks.^182^ 10% of Mg^2+^ can be used in conjunction with 10% type I fibrillary collagen gel and 80% HA to decrease the degradation of these composites up to 21 days.^183^ Porous HA scaffolds coated with type I collagen cross-linked using *N*-(3-(dimethylamino)­propyl)-N′-ethylcarbodiimide hydrochloride (EDC) and *N*-hydroxysuccinimide (NHS), and incorporated with heparin, were developed for the controlled release of BMP-2, that were sustained for 7 days in vitro.^184^

**Figure 13:**
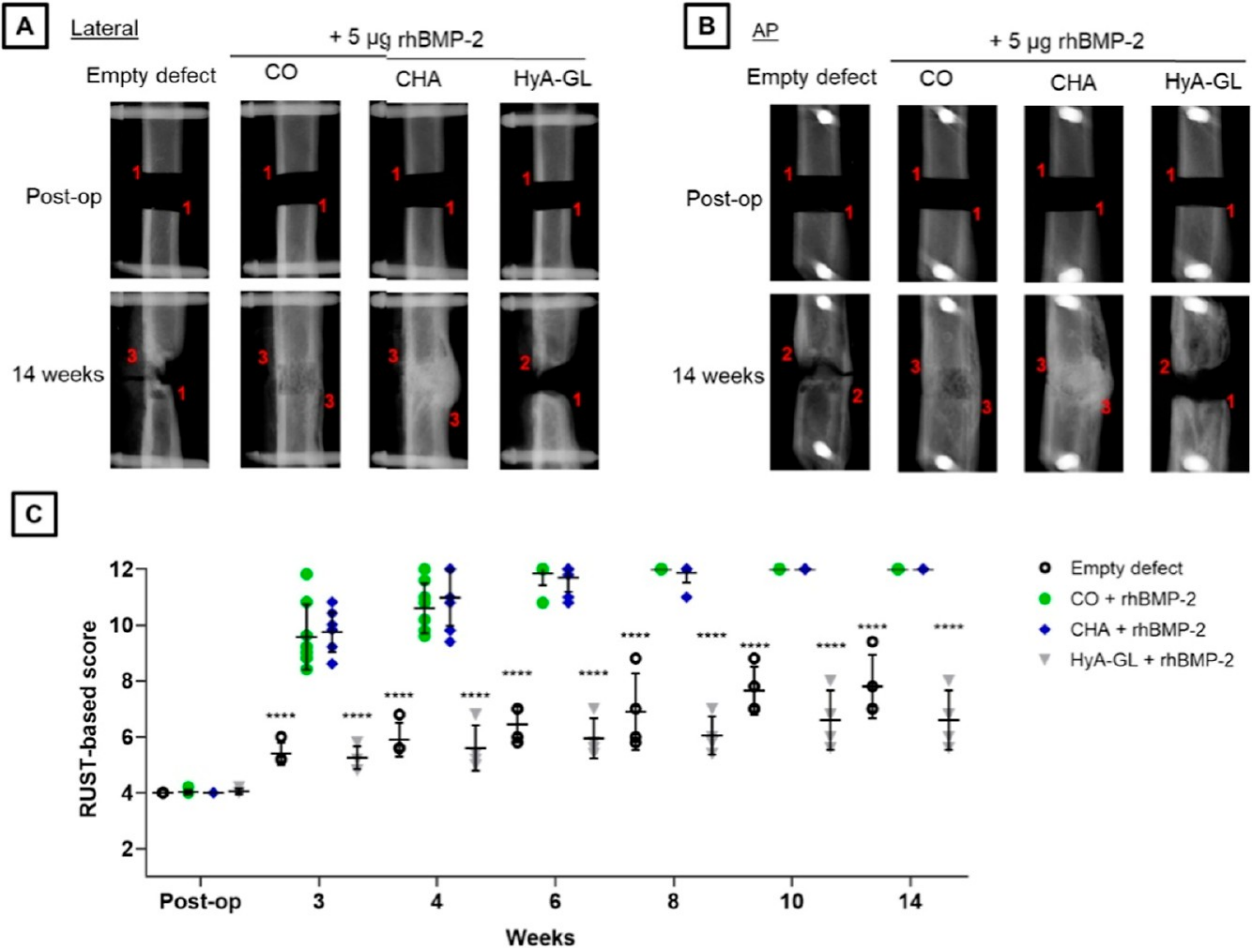
RUST-based assessment of cortical bridging. Representative digitally reconstructed radiographs (DRRs) from each group are shown in (a) lateral, and (b) anteroposterior (AP) view. An example score (1–3) is given to the two cortices in each DRR, creating a maximum possible RUST-based score of 12 when considering lateral and AP DRRs of the same animal. (c) Average RUST-based scores for each animal were plotted as a function to show the progression of cortical bridging over time. “Reproduced with permission from.^177^ Copyright (2021) Elsevier.”

The combination of poly­(lactic-*co*-glycolic) acid (PLGA), type I collagen, and HA was also employed to create multilayered 3D scaffolds through electrospinning, which controlled the adhesion, proliferation, and osteogenic differentiation of MC3T3-E1 cells.^185^ PLGA scaffolds and collagen-chitosan-HA hydrogels were implanted for 14 days in the dorsal skin chambers of mice to study angiogenic and inflammatory responses. The PLGA scaffolds demonstrated a slight increase in leukocyte recruitment and microvascular permeability while promoting the formation of new capillaries. In contrast, the hydrogels induced severe inflammation and failed to support new vessel growth, indicating that PLGA can improve biocompatibility and favor vascular growth.^186^ Hydrogels made of 4, 7, 10 and 15% poly­(vinyl alcohol) (PVA), type I collagen, and HA were also found to be biocompatible and nontoxic to MC3T3 preosteoblasts, with reinforcement from HA and collagen reducing the inflammatory response from RAW 264.7 macrophages.^187^ Additionally, they exhibited great potential as drug delivery systems, particularly for the release of erythromycin. Chang et al. designed biohybrid gel beads in ratio of 35:65 type I COL/HA. This bead was filled in a ringed polytetrafluoroethylene (PTFE) artificial vascular graft and implanted in iliac crest of New Zealand rabbits.^164^ Observations showed that these scaffolds, combined with the local injection of autologous platelet-rich plasma, significantly increased the area (61.83%) and density of calcification (0.95%) with a degradation of the beads and new tissue formation with capillaries and osteoid around matrixes.^164^

HA and collagen coatings on PLGA and β-TCP (7:3 ratio) scaffolds improved BMSCs cell proliferation and ALP activity while increasing their hydrophilicity compared to uncoated scaffolds after 14 days.^188^ Cell adhesion and proliferation of MC3T3 cells, along with enhanced mechanical properties of 15% PVA fibers, were also achieved by encapsulating 10% HA and 10% collagen within these fibers, making them excellent candidates for orthopedic prosthetic surfaces.^189^ Nanofibers of type I collagen and nanophased HA using the electrospray technique promoted the growth of human dermal neonatal fibroblasts (HDNF), HaCaT keratinocytes and hMSCs cells, proliferation, differentiation, and increased extracellular matrix production through calcium ions release.^190^ Furthermore, this material was able to prevent the adhesion of pathogenic bacteria found in human skin flora. Uezono et al. coated titanium discs with HA and collagen (80:20 ratio), observing that all implants were almost completely surrounded by new bone tissue without encapsulation, exhibiting the greatest bonding strength to bone within 4 weeks.^191^ HA obtained from *Pinctada maxima* shells, combined with bovine collagen and deposited by electrophoresis on titanium discs, also showed high apatite growth after immersion in simulated body fluids.^192^

Among the alternative materials for collagen scaffolds, TiO_2_ nanoparticles demonstrate significant potential. Titanium is already a widely used material for implants, particularly orthopedic, due to its low density and excellent corrosion resistance. The addition of titanium to proteins like collagen can increase material strength, elasticity, and fracture toughness.^193^ Elango et al. created a matrix of collagen, sodium alginate, and TiO_2_ nanoparticles capable of treating periodontitis (Figure 14).^194^ The combined scaffold supports and stimulates osteogenesis, accelerating the development of collagen, alkaline phosphatase, and osteocalcin through a Runx2 signaling mechanism. The material exhibited suitable parameters such as stiffness, swelling, contraction factor, and osteogenic differentiation for periodontal regeneration applications. Exploring the current context and the already developed and applied TiO_2_, its integration into protein structures offers a great advantage. This oxide is one of the most promising nanoparticles due to its biocompatibility combined with anticancer and antimicrobial properties. Li et al. explored 3D scaffolds made from a mixture of type I collagen from porcine skin and TiO_2_ nanoparticles coated with polyvinylpyrrolidone (PVP).^195^ These compounds are linked by four main hydrogen bonds necessary for scaffold formation, resulting in a biomaterial with higher tensile strength and lower elongation. The material proved promising in wound healing due to the acquisition of adequate mechanical properties from the addition of nanocomposites, making it a simple yet effective skin regenerator. The fabrication of scaffolds with TiO_2_ nanoparticles, although quite common and advantageous, has also been studied in combination with other materials. As reported by Vedhanayagem et al., a collagen structure grafted with poly­(methyl methacrylate) (PMMA) was reinforced with PdO–TiO_2_ nanocomposites.^196^ The choice of PMMA for modifying the base structure was due to its thermal stability and nontoxicity, already used in hip and knee replacements, while the palladium structure was selected for its pro-drug activation and antimicrobial activities. Consequently, g-PMMA-Collagen/PdO–TiO_2_ (1:1 mol ratio) scaffolds, compared to collagen-only materials, exhibited greater mechanical strength (1491 ± 20 kPa–945 ± 16 kPa), nontoxicity, and enhanced in vitro osteogenic activity in human osteosarcoma MG 63 cells, without affecting the material’s conformation or biocompatibility, making it a viable option for skin tissue engineering.

**Figure 14:**
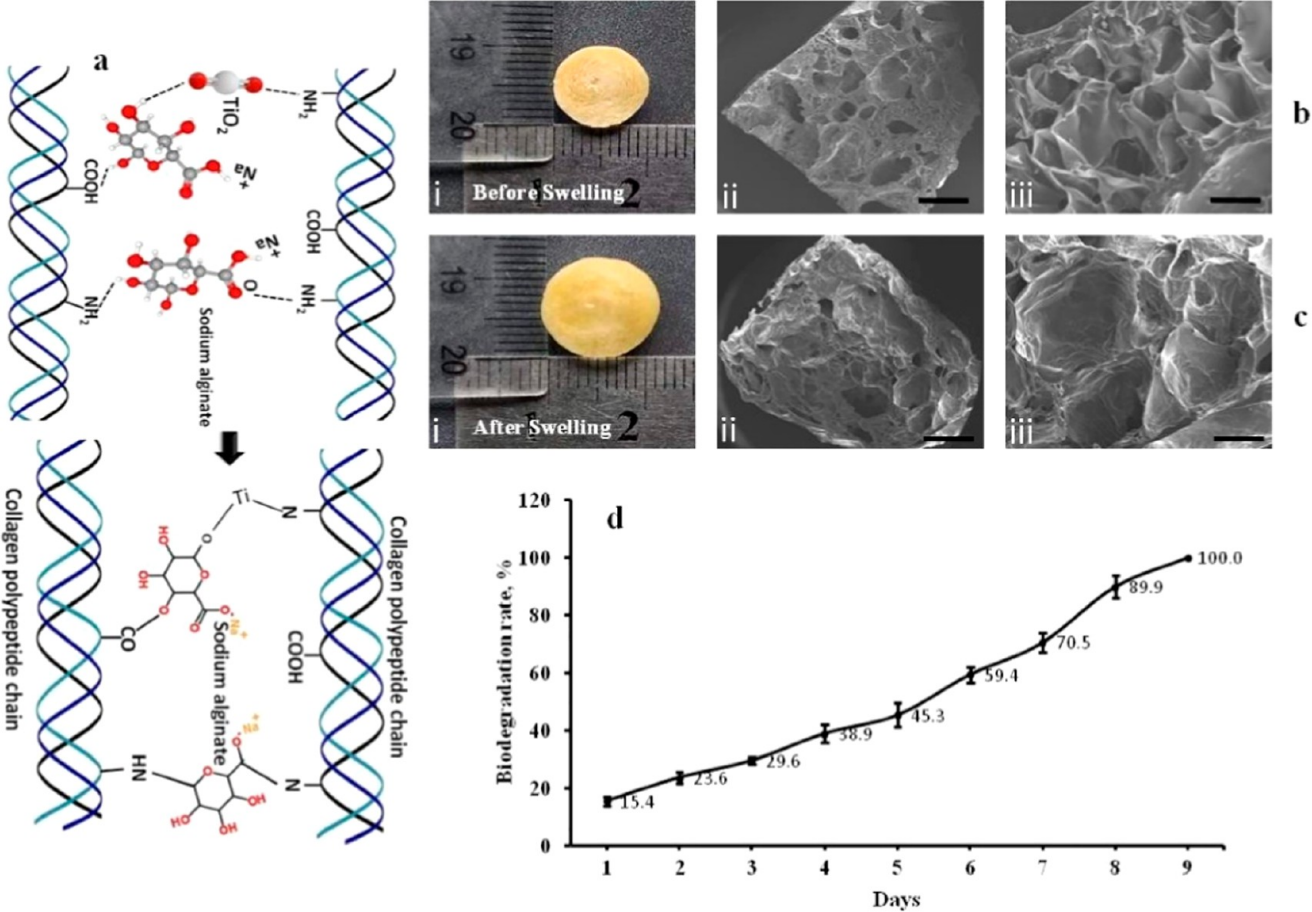
(a) Schematic representation and (b) Microstructural changes before and (c) after Swelling and (d) in vitro biodegradation of the 3D matrix. “Reproduced with permission from.^191^ Copyright (2020) Elsevier.”

Collagen-only scaffolds have lower mechanical strength and rapid biodegradation, but when metal oxide cross-linkers are used, the material’s stability increases. This fact is again reported by Vedhanayagam et al., who analyzed scaffolds of different oxides and observed that for skin regeneration applications, the best nanoparticles were ZnO > TiO_2_ > CeO_2_ > SiO_2_ > Fe_3_O_4_.^197^ This result was directly related to greater mechanical strength (273.14 ± 0.2 MPa Young’s modulus), cell viability, and better antimicrobial activity of the 14 nm of ZnO-TES-PAMAM-G3 collagen scaffold (collagen scaffold cross-linked with metal oxide nanoparticles functionalized with triethoxysilane-poly­(amidoamine) generation 3). In this case, the nanoparticles modified the physical, optical, magnetic, and antibacterial properties, while the collagen coating served as a skin substitute. In working with collagen cross-linking to increase its stability, Agban et al., performing a cross-linking of ZnO nanoparticles coated with PVP, noticed that as the concentration of the cross-linked material increased, stronger scaffolds with more stable drug release were formed.^198^ The increased concentration of cross-linked material was responsible for greater hardness (3.10 ± 0.42 g), adhesiveness (2.7 ± 0.3 g·s^–1^), rheological properties (G′ than G″), and better mechanical properties, allowing the biomaterial’s different applications in tissue engineering. The application of ZnO stands out mainly due to the modulation of scaffolds mechanical properties, due the semiconductor, catalytic and piezoelectric properties of ZnO.^199^ Balaure et al. studied the action of new dressings based on ZnO nanoparticles functionalized with collagen and essential oil, primarily applied in wound dressings, especially burns, and in reducing sepsis development (Figure 15).^200^ The fabricated material showed good biocompatibility, noncytotoxicity, antimicrobial potential, and bioabsorbability, accelerating wound healing, making it an easy-to-use and apply scaffold with potential for the production of customized multifunctional dressings.

**Figure 15:**
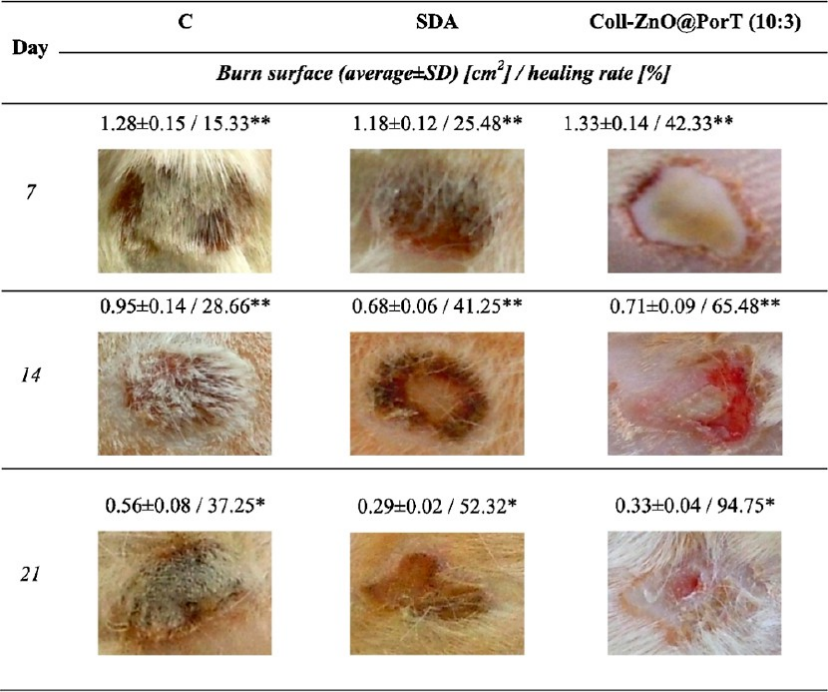
Macroscopic evolution of wounds after the application of the dressings (C): Control group with spontaneous healing; (SDA): Control group treated with 1% silver sulfadiazine cream; Coll-ZnO@PorT: The group to which the nanocomposite dressing was applied; SD: Standard deviation; *p < 0.01; **p < 0.05. “Reproduced with permission from.^197^ Copyright (2019) Elsevier.”

The use of bioactive glass (BG) in the CaO–P_2_O_5_–SiO_2_ composition is an attractive and well-known material in literature as an inorganic material for tissue engineering, especially bone tissue. Sarker et al., aware of collagen lack of stability in its pure form, combined it with BG.^201^ The use of BG was able to increase the scaffold compressive strength and structural rigidity, in addition to allowing controlled drug delivery. Therefore, due to its ability to enhance osteogenesis, odontogenesis, and angiogenesis, the scaffold becomes promising for bone tissue engineering. The combination of collagen and BG, as reported by Caddeo et al., aims to improve the scaffold by creating a material capable of mimicking the organic and inorganic phases of the bone extracellular matrix (BEM).^202^ In this work, collagen mixed with water-soluble polyurethane was obtained for exposure of the amino groups of the polymer chain, which can be exploited for stabilization through cross-linking processes. Cross-linking with a bioactive vitroceramic SiO_2_–P_2_O_5_–CaO–MgO–Na_2_O–K_2_O substrate provided a good substrate for bone cell adhesion and growth in MG-63 cell line up to 21 days, capable of mimicking the nature of the bone BEM. The treatment of bone disorders and defects is challenging due to its structural complexity. Dhinasekaran et al. fabricated three-dimensional membranes composed of inorganic BG fibers with organic collagen structure to generate a synthetic bone substitute.^203^ BG had a significant influence on ideal hydrophilicity, bioactivity, and in situ drug delivery. The final scaffold enhanced cell adhesion and proliferation due to its proximity to the natural BEM. Furthermore, Runx2, Col-Type-1 mRNA, osteocalcin, and osteonectin levels were significantly increased in cells cultured using the formed scaffold, making the 3D multifunctional membrane applicable for treating bone defects. Other works related to the application of scaffolds for bone tissues have been reported, such as Hsu et al., where mesoporous BG nanofibers (MBGNFs) formed scaffolds associated with collagen (CM) using electrospinning techniques.^204^ When in contact with body fluid, apatite minerals formed, providing a suitable environment for cytoskeleton attachment. Additionally, the scaffold promoted differentiation and mineralization of MG63 osteoblast-like cells, and a higher bone regeneration in vivo model of calvaria defect in rats.

Moreover, in addition to enhancing the application of collagen in tissue regeneration, the incorporation of metal oxide nanoparticles for magnetic processes is highly regarded in the field of tissue engineering and imaging diagnostics. As reported by Nidhin et al., the cross-linking of collagen with Fe_2_O_3_ nanoparticles coated with amine was found to be nontoxic, with increased thermal stability, improved paramagnetic properties, enabling its application as a fluorescent probe, and in magnetic resonance imaging, thus allowing the use of scaffolds as bioimplants and in imaging diagnostics via fluorescent nanonetworks.^193^ Regarding scaffolds for imaging diagnostics, Mertens et al. focused on the labeling of cells with ultrasmall superparamagnetic Fe_2_O_3_ nanoparticles (USPIO) through a chemical cross-linking process with collagen from porcine tissue.^205^ This incorporation was optimized and correlated with signal intensity in magnetic resonance imaging, demonstrating the high biocompatibility of the scaffolds formed. These markers make it possible to noninvasively visualize the location, resorption, and function of tissue engineering implants. However, the use of Fe_2_O_3_ nanoparticles through a chemical cross-linking process with collagen is not limited to imaging diagnostics. By studying the influence of paramagnetic Fe_2_O_3_ nanoparticles coated with polyethylene glycol and suspended in collagen, Bonfrate et al. explored the formation of a scaffold with adjustable electrical conductivity that was stable over 15 days and promoted an increase in the viability (99%) of 3T3 cells after 48 h.^206^ Although the material application was not analyzed, it allowed the development of mechanisms for the differentiation of stem cells from a given lineage.

Oxide-based nanoparticles have proven to be among the most versatile and functionally diverse additives for collagen scaffolds, bridging the gap between mechanical reinforcement, bioactivity, and multifunctionality. Hydroxyapatite remains the benchmark material for bone regeneration due to its compositional similarity to natural bone and strong osteoconductive potential, yet the variability in porosity, cross-linking, and degradation control still challenges consistency across studies. TiO_2_ and ZnO extend these capabilities, adding photoreactive, catalytic, and piezoelectric functionalities that improve mechanical strength and antimicrobial performance but can also generate reactive species capable of disturbing cell homeostasis if not carefully regulated. Si- and Mg-based oxides further contribute to mineralization and cell signaling, while iron oxides and bioactive glasses introduce imaging contrast, magnetically guided regeneration, and controlled ion release, though at the cost of increased compositional complexity and potential stability trade-offs. Despite these advances, reproducibility remains a critical limitation: minor changes in nanoparticle synthesis, surface modification, or collagen source can significantly alter biological outcomes. Moreover, the long-term effects of ion release, ROS generation, and the cumulative exposure of surrounding tissues are still insufficiently characterized. To advance oxide–collagen composites beyond experimental success, future research must couple precise control of nano–bio interfaces with standardized fabrication and evaluation protocols, integrating mechanical, chemical, and biological data to predict scaffold behavior under physiological conditions.

## 5 Trends and Perspectives

Collagen remains the protein of choice when biocompatibility and cell–matrix signaling matter, but on its own it is mechanically weak and degrades too quickly. Pairing collagen with inorganic fillers changes the equation. Carbon materials build load-bearing and conductive networks that couple mechanical reinforcement with electrical cues. Metallic nanoparticles act as local reservoirs of antimicrobial and pro-regenerative ions. Oxides supply mineral phases and stimuli-responsive behaviors that collagen lacks. Used judiciously, the composite becomes more than the sum of its parts: it carries load, resists enzymes, guides cells, and releases signals on demand.

From the growing literature, several design insights emerge. First, architecture is as decisive as chemistry. Aligned pores from directional freezing, printed lattices, and electrospun fibers not only expand surface area but dictate nutrient transport and cell orientation. Second, interfaces govern performance. Gentle cross-linking and polymer grafts stabilize the inorganic–organic junction, minimize swelling, and confine ion or drug release within therapeutic limits. Third, functionality must remain within narrow windows. Moderate conductivity sustains bioelectric signaling without thermal or faradaic damage, and steady low-dose ion flux supports angiogenesis or osteogenesis without triggering cytotoxicity.

Nevertheless, the path to consistency is far from straightforward. Experimental outcomes still vary widely due to differences in collagen origin, fibrillogenesis control, particle morphology, and surface functionalization. These variables complicate direct comparisons between studies and make it difficult to establish generalizable design rules. From a safety perspective, long-term behavior remains particularly underexplored, as most available evidence relies on short-term in vitro release assays or simulated biological fluids, which cannot fully capture the complexity of in vivo clearance and biodistribution processes. Long-term safety remains poorly mapped: few studies trace the fate of nanoparticles, their chemical evolution, or macrophage responses months after implantation. Systematic investigations addressing metal accumulation, transport through lymphatic pathways, and eventual elimination routes are still scarce, limiting robust conclusions on chronic exposure risks.^132^ Additionally, sterilization protocols often compromise biological or electronic functionality, revealing a gap between laboratory performance and clinical readiness. The multifunctional nature of these devices also blurs the line between drug and implant, demanding early regulatory planning and interdisciplinary oversight.

Even so, several near-term paths look realistic:

- Trimodal scaffolds for hard tissues: Constructs that combine conductive networks for cell alignment, mineral-like phases for structural support, and antimicrobial elements in low doses, organized in gradients that mirror the natural transition from soft to mineralized tissue.
- Stimuli-responsive wound dressings: Matrices designed to trigger antimicrobial activity when exposed to external cues such as light or mechanical stimulation, reducing the reliance on systemic antibiotics while protecting healthy tissue.
- Angio-centric bone repair: Scaffolds tailored to encourage early microvascular growth, integrated with controlled release systems that deliver osteoinductive factors in safe and effective microdoses.
- Magnetically assisted implants: Magnetic materials–collagen matrices that can be imaged, guided, or gently heated for hyperthermia-aided healing, while maintaining high biocompatibility.
- Data-guided formulation: High-throughput variations in particle loading, cross-link density, and pore anisotropy mapped to mechanical, electrical, and biological readouts to shorten iteration cycles.

Longer term, the field is likely to converge on a few principles: fewer components, better characterized. Rather than stacking many additives, lean formulations will target clear clinical end points—rapid infection control, stable vascularization, durable mechanics, and restored function—while surviving sterilization and shelf life. Integrated sensing and closed-loop actuation are within reach: collagen–carbon conductors that report local pH or ROS and deliver electrical or drug pulses only when needed. On the biological side, immune-instructive surfaces that bias macrophages toward pro-healing phenotypes will be as important as osteogenic or neurogenic signals. Finally, sustainable sources of collagen (e.g., marine) and on-demand manufacturing (printing or molding at the point of care) will improve access and consistency.

Yet, translation remains the central bottleneck. Preclinical success frequently falters at the transition to scale, where uncontrolled batch variation, unpredictable degradation kinetics, and limited long-term biocompatibility data impede regulatory acceptance. The multifunctional nature of these composites—structural, antimicrobial, and bioelectronic—challenges existing classification systems, demanding updated standards and coordinated testing frameworks. Moving forward, success will depend less on incremental material tweaks and more on reproducible fabrication, quantitative validation, and sustained collaboration between materials scientists, clinicians, and regulatory bodies. Only through this integration can collagen–inorganic composites mature from promising prototypes into reproducible, clinically reliable platforms for regenerative medicine.

## Data Availability

No new data were created or analyzed in this study. Data sharing is not applicable to this article.
